# Feasibility studies of multimodal nonlinear endoscopy using multicore fiber bundles for remote scanning from tissue sections to bulk organs

**DOI:** 10.1038/s41598-023-40944-6

**Published:** 2023-08-23

**Authors:** Hyeonsoo Bae, Marko Rodewald, Tobias Meyer-Zedler, Thomas W. Bocklitz, Gregor Matz, Bernhard Messerschmidt, Adrian T. Press, Michael Bauer, Orlando Guntinas-Lichius, Andreas Stallmach, Michael Schmitt, Juergen Popp

**Affiliations:** 1 Leibniz Institute of Photonic Technology (Leibniz IPHT), Member of Leibniz Health Technologies, Member of the Leibniz Centre for Photonics in Infection Research (LPI), PO Box 100239, 07702 Jena, Germany; 2 Institute of Physical Chemistry and Abbe Center of Photonics, Friedrich-Schiller University Jena, Helmholtzweg 4, 07743 Jena, Germany; 3 Center for Sepsis Control and Care (CSCC), Jena University Hospital, Erlanger Allee 101, 07747 Jena, Germany; 4 GRINTECH GmbH, Schillerstraße 1, 07745 Jena, Germany; 5 Department of Anesthesiology and Intensive Care Medicine, Jena University Hospital, Am Klinikum 1, 07747 Jena, Germany; 6 Medical Faculty, Friedrich-Schiller University Jena, Kastanienstr. 1, 07747 Jena, Germany; 7 Department of Otorhinolaryngology, Jena University Hospital, Am Klinikum 1, 07747 Jena, Germany; 8 Department of Internal Medicine IV, Jena University Hospital, Am Klinikum 1, 07747 Jena, Germany

**Keywords:** Physical chemistry, Raman spectroscopy, Optics and photonics, Biophotonics

## Abstract

Here, we report on the development and application of a compact multi-core fiber optical probe for multimodal non-linear imaging, combining the label-free modalities of Coherent Anti-Stokes Raman Scattering, Second Harmonic Generation, and Two-Photon Excited Fluorescence. Probes of this multi-core fiber design avoid moving and voltage-carrying parts at the distal end, thus providing promising improved compatibility with clinical requirements over competing implementations. The performance characteristics of the probe are established using thin cryo-sections and artificial targets before the applicability to clinically relevant samples is evaluated using ex vivo bulk human and porcine intestine tissues. After image reconstruction to counteract the data’s inherently pixelated nature, the recorded images show high image quality and morpho-chemical conformity on the tissue level compared to multimodal non-linear images obtained with a laser-scanning microscope using a standard microscope objective. Furthermore, a simple yet effective reconstruction procedure is presented and demonstrated to yield satisfactory results. Finally, a clear pathway for further developments to facilitate a translation of the multimodal fiber probe into real-world clinical evaluation and application is outlined.

## Introduction

Label-free in vivo imaging of tissues providing both morphological and chemical information is crucial for many envisioned medical applications, particularly for an intraoperative non-invasive histopathologic examination of tissue. Within the last years, it has been shown that combining different spectroscopic techniques in a multimodal imaging approach is beneficial to meet all requirements for speed, depth of penetration, and molecular specificity^1–3^. One such approach is Coherent Anti-Stokes Raman Scattering (CARS) microscopy, which simultaneously co-generates the two other non-linear effects, Two-Photon Excited Fluorescence (TPEF) and Second Harmonic Generation (SHG), in a single imaging device. CARS enables mapping a specific molecular vibration, with the most frequently chosen ones being indicative of predominantly lipids (e.g., ~ 2855 cm^−1^, ν_s_(CH_2_)) or proteins (e.g., ~ 2930, ν_s_(CH_3_)), both of which are abundant in biological samples. In contrast, TPEF can be used to address endogenous auto-fluorophores, most notably NAD(P)H, which is omnipresent in tissues due to its importance for cell metabolism. Furthermore, SHG is a process only occurring in non-centrosymmetric materials, making it highly specific for quasi-crystalline biomaterials like collagen fibers or myosin filaments. Thus, combining those three non-linear modalities provides valuable insight into a tissue’s morphochemistry in a label-free manner.

In this context, we have demonstrated that multimodal non-linear microscopy combining CARS, SHG, and TPEF enables the detection of characteristic structures and the accompanying molecular changes of widespread diseases, particularly cancer^4,5^. To facilitate interpreting CARS/SHG/TPEF image data, advanced image processing algorithms can automatically extract characteristic properties^6,7^. In addition and alongside automatic evaluation, it could be shown that the information encoded in these label-freely recorded multimodal images can also be translated into computational hematoxylin and eosin (H&E)^8^ images by multivariate statistics, which not only taps into the existing body of knowledge and training of medical professionals but might also help transitional acceptance. To generate such computational H&E images and/or to provide an automated evaluation of multimodal nonlinear imaging data—including, disease grading or visual segmentation as a basis for further clinical decision making—on-site and directly during surgery, compact hand-held endoscopic devices are required. With application scenarios ranging from tumor margin detection in surgical wounds to symptom investigation and disease detection, grading, and monitoring in hollow organs (e.g. inflammatory bowl disease^9^), the development of endoscopic devices for non-linear spectroscopic imaging has been a subject of significant interest for many years. Different approaches have been presented: besides point scanning probes^10^, the most common ones are scanning fiber endoscopes^11–18^ and using galvo scanning mirrors or microelectromechanical system (MEMS) scanners^19–24^.

While point scanning probes do not provide spatial information, the other types of devices contain delicate moving optomechanical parts making them susceptible to misalignments and operating failures in the long term, thereby negatively impacting their reliability and making regular maintenance by qualified personnel necessary. The adverse conditions of typical sterilization procedures, such as autoclaving, are of particular concern in this regard, as are mechanical forces that are likely to arise during general handling. The use of scanning devices in the probe head can be avoided by utilizing multi-core imaging fibers for delivering the excitation laser pulses. These imaging fibers, like the FIGH-10-500N from Fujikura Ltd. Japan, consist of several thousand coherent light-guiding elements, which preserve the spatial relationship between the two fiber ends. It was demonstrated that these fiber bundles can be used for imaging purposes by shifting the laser scanning procedure from the distal to the proximal end of the fiber^25–30^. Previous work has demonstrated that also CARS imaging is possible using such fiber bundles^31^ and that integration into a compact fiber probe is possible by utilizing gradient index (GRIN) optics^32^. However, to mitigate temporal and spectral broadening, it is recommended to employ a picosecond lasers as the excitation source. The dispersion characteristics associated with gradient-index (GRIN) optics and optical fibers, particularly when utilized with a femtosecond laser, have been thoroughly examined and can be found in the cited literature in^33^. In addition to the anticipated benefits regarding the long-term stability of such a probe, the all-optical design avoids any current-carrying parts in the probe head, whereas the above-mentioned scanning approaches require high voltages, mandating the evaluation of potential risks for the patient and likely complicating approval of the technology.

In spite of the obvious appeal of this approach, to our knowledge, previous work^32^ in our group still represents the only published example of a coherent fiber bundle-based endoscope that demonstrated simultaneous recording of CARS, TPEF, and SHG. While TPEF and SHG are markedly easier to implement, CARS—despite it being a particularly desirable modality for the analysis of biological samples—has remained challenging. However, with the focus of these previous studies^31,32^ being mostly on the technical realization, characterization aspects, and some low-fidelity proof-of-concept images of thin tissue sections, questions regarding the general applicability to real-world samples under practical aspects, the achievable image quality, and suitable data processing routines, remain insufficiently addressed. In this contribution, an improved probe design and data analysis strategy are presented, enabling the application to medically more relevant samples and scenarios.

After a brief review of the probe design including its technical improvements, first, general performance characteristics, particularly concerning optical resolution, are established. Using a dried thin section of human head and neck cancer tissue as a well-defined biological, flat sample that provides contrast in all three channels, conformity with LSM (laser-scanning microscope)-based measurements is analyzed. Then, a series of measurements of polymer beads of varying sizes are presented to assess the optical resolution and its limitations due to sparse sampling. The resolution limits if sparse sampling by individual fiber cores is counteracted by taking multiple spatially shifted measurements are analyzed. These findings are tested on a thin section of murine liver tissue. With the performance characteristics established, the central part of the presented study concentrates on two aspects: image reconstruction to provide readily human-readable images and the probe’s applicability to bulky samples more representative of the envisioned application scenario of in vivo measurements for on-site spectral histopathology. For this purpose, porcine and human intestinal wall segments are investigated ex vivo as realistic, medically relevant samples.

## Characterization of the probe

### Probe design

**Figure 1:**
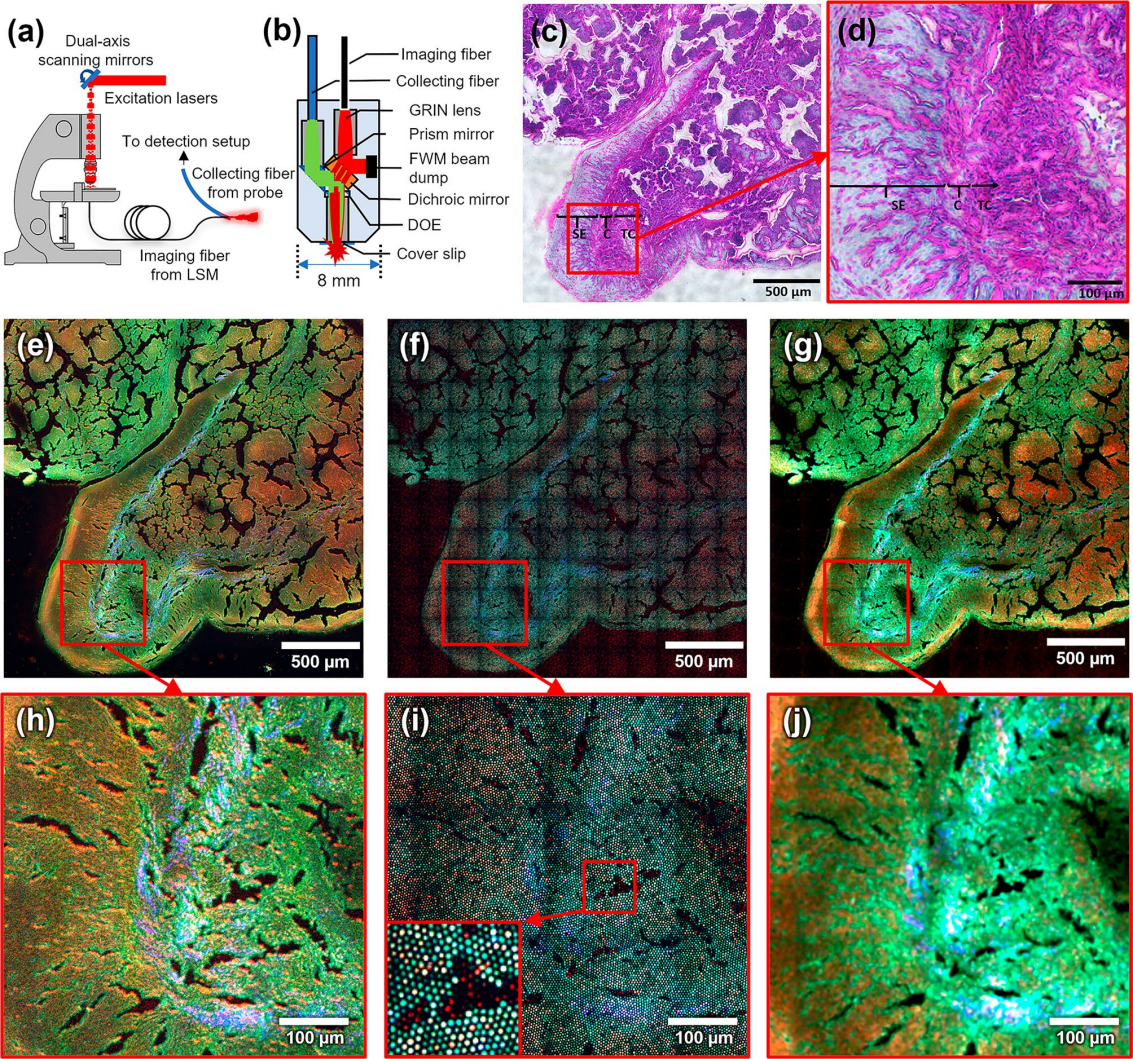
(**a**) Experimental setup with a laser scanning microscope coupled to the probe. The excitation lasers are coupled into the imaging fiber using a laser scanning microscope. The light is transferred by the imaging fiber and focused in front of the probe head, where the non-linear signal is generated within the tissue sample. The signal is collected by a multimode fiber and guided to a detection setup with three simultaneous detection channels. (**b**) Internal fiber probe design. (**c**) H&E-stained image of a head and neck cancer tissue. Assignment: squamous epithelium (SE), connective tissue (C), and region of tumor cells (TC). (**d**) Enlarged and detailed view of the area marked in (**c**). (**e**) Multimodal image of the same region recorded with a conventional laser scanning microscope. (**f**) Merged series of the unprocessed fiber probe images of the same region. (**g**) The same image shown in (**f**) after applying an image processing algorithm presented in the image processing section. (**h–j**) Enlarged and detailed view of the areas marked in (**e**), (**f**), and (**g**), respectively. Color coding: red—CARS, blue—SHG, green—TPEF. Tile size in fiber probe images: 207 × 207 µm^2^.

The fiber probe and the general experimental setup including light sources, LSM, and the three-channel detection setup, have been described in great detail elsewhere^32^. The key element of the fiber probe is the coherent imaging fiber consisting of ~ 10,000 individual cores (FIGH-10-500N, Fujikura Ltd. Japan) for delivering the excitation laser beams, together with an image field- and color-correcting optical system. Here, we applied a refined probe design with technical improvements in terms of laser illumination and signal collection efficiency. While previous studies focused on CARS, to ensure optimal performance also in the TPEF and SHG channel for the purposes of this work, the previously employed longpass filter (LP02-830RE, Semrock, USA) was exchanged for a dichroic beam splitter (Di02-R785) which provides higher transmission (> 90%) in the wavelength range of 400–780 nm. The GRIN lense and DOE assembly was manufactured to stricter tolerances, minimizing vignetting and chromatic errors of the pump and Stokes beams. Additionally, the probe head was refined with a new metal housing to protect and enforce the otherwise fragile fiber connection. For the same reason, Medical Device Regulation (MDR) approved endoscope tubes and SMA connectors were applied to the fibers. A home-built LSM has been used for coupling the excitation laser pulses generated by an 80 MHz mode-locked Nd:VAN laser (picoTRAIN, High Q Laser, Austria) in combination with an optical parametric oscillator (Levante Emerald, A.P.E, Germany) at 816 nm (pump) and 1064 nm (Stokes) into the imaging fiber^34^. This wavelength pair corresponds to a Raman resonance of 2850 cm^−1^, matching the symmetrical stretching vibration of CH_2_ groups particularly abundant in lipids, which results in a CARS signal at 661.8 nm. As excitation wavelength for the SHG modality, we used the 1064 nm beam, while both beams served as the excitation source for the TPEF modality. The proximal end of the imaging fiber is placed in the focal plane of the LSM where it is scanned by two galvo mirrors in a dense raster pattern, while the distal end of the probe is placed at the tissue sample. The full image circle diameter measures ~ 460 µm, however, a reduced scanning field (~ 260 µm) in the central region of the imaging fiber is used to avoid damaged cores in the fiber, which might have been caused during the manufacturing process of the probe head. The power of the laser beams at the sample site was about 50 mW for pump and 25 mW for Stokes in the probe measurement and 70 mW for pump, and 40 mW for Stokes in LSM recordings. The average laser power used for nonlinear endoscopic imaging provided sufficient signal generation from the presented samples without causing visible damage to any of them and aligns with the power scales utilized in comparable nonlinear endoscopic imaging applications^18,35^ with use of picosecond laser pulses. A useful point of reference in this context is provided by Galli et al.^36^ who investigated photodamage under similar excitation conditions as a function of recorded frame repetitions. A schematic setup of the LSM coupled to the probe is depicted in Fig. 1a. Figure 1b shows the internal optical design of the probe head. Two notch dichroic beamsplitters (NFD01-532 (Semrock, USA) and F73-067 (Chroma, USA)), and three bandpass filters (FF01-661/20 (Semrock, USA), FL532-10 (Thorlabs, USA) and FF01-550/88 (Semrock, USA)) were used in addition to a short-pass filter (FESH0700, Thorlabs, USA) to separate sample signals. In the current design iteration, all optics are designed for a measurement through 170 µm of glass. For easier prototyping and more versatility in the testing phase, the current design does not include a fixed glass window and instead relies on a cover glass to be placed on the sample. The probe design is largely agnostic to the specific LSM used and could, for example, be coupled with a compact fiber-based laser system and scan head to be incorporated into a mobile station, as demonstrated recently for a rigid-body nonlinear endoscope^35^. An overview of key structural and performance characteristics of the probe is given in Table 1.

**Table 1:** Key structural and performance characteristics.

Probe length	32 mm
Probe diameter	8 mm
Fiber diameter |+ coating	~ 500 µm |~ 600 µm
Imaging diameter	~ 460 µm
# fiber cores	~ 10,000
Core-to-core distance (avg., measured)	4.69 µm
Mean core diameter	2.9 µm
Wavelengths (incupling)	816 nm, 1064 nm
Resolution^a^ | spot size^b^	10.6 µm | 3.3 µm
FOV (inscribed square, theor.)	325 µm × 325 µm
Pixel dwell time (practical)^c^	2 µs
Peak signal to noise ratio (single frame)^d^	
CARS	50
TPEF/SHG	10
Transmission (whole system)	25% (pump)
	26% (Stokes)
Pulse width proximal | after 1 m fiber	3.3 ps | 3.7 ps pump
	9.1 ps | 9.8 ps Stokes
r_bend_ (stiffer fiber, long | short term)	9.6 cm | 4.8 cm

^a^2.3 × core-to-core center distance, ^b^simulated FWHM of spot of idealized point source in object plane (1.9 µm) convoluted with mean core diameter, ^c^@256 × 256 px, 4 frame averages: 2 images/s (see also Fig. S3), ^d^calculated for Fig. 1j for a single frame, see SI for details.

### General performance

To demonstrate the performance of the refined probe as an endoscopic imaging device, a human head and neck cancer tissue section (20 µm, air-dried native cryosection) was imaged. The probe was placed directly above the thin tissue section while a motorized stage moved it. Figure 1f shows a merged multimodal image series (12 by 12 tiles, 190 µm lateral shift) without any post-processing applied except for slight brightness adjustments for proper visualization. Images were significantly oversampled to provide maximum flexibility in the data processing and a baseline of achievable image quality. The acquisition time for a single tile was 32 s at a digital resolution of 2048 by 2048 pixels with a pixel dwell time of 2 µs and four times frame averaging. Under some assumptions and approximations, the peak signal-to-noise-ratio for a single frame can be estimated to be 52 for CARS and 11 for TPEF/SHG (see SI for details). Compared to the previous results^32^, the refined probe design shows a significantly higher signal collection efficiency for all three non-linear modalities. The multimodal image shown in Fig. 1f reflects the morpho-chemical structure of the human head and neck cancer tissue composed of pronounced CARS from lipid-rich structures, TPEF from endogenous autofluorophores (elastin, NADH, FAD), and SHG from collagen fibers. The morphology of the tissue correlates well with an H&E-stained image of the same tissue section, including squamous epithelium (SE), connective tissue or stroma (C), and regions with tumor cells (TC) in Fig. 1c,d. We were able to acquire CARS and TPEF signals in the area of epithelium corresponding to lipids and elastin, which can be seen in the rest of the tissue as well. Moreover, collagen and elastin fibers, as a combination of SHG and TPEF signals (seen in light blue color), can be nicely visualized within the area of connective tissue confirmed by H&E staining. The same region investigated by the probe was recorded by a conventional LSM (Fig. 1e) with the same scanning parameters described above for direct comparison, with the only difference being a lateral shift of 550 µm to account for a larger field of view. It can be seen, that the observed morphochemistry within the LSM image (Fig. 1e) corresponds well with the one recorded with the fiber probe (Fig. 1f). However, the image recorded with the probe, as compared to the LSM image, shows a rather pixelized structure with lower spatial resolution due to the multi-core composition of the imaging fiber (see Fig. 1e,f). An image processing algorithm counteracting the pixelization (which will be described later in great detail) was developed and applied to correct the pixelated structure. The result is shown in Fig. 1g. It can be seen that the fiber probe design combined with appropriate image processing algorithms produces a multimodal non-linear image of the thin tissue section with image quality comparable to that obtained with the conventional LSM. Noteworthily, tumorous regions appear notably brighter in the TPEF channel than surrounding healthy regions due to increased autofluorescence, which is consistent with earlier reports for head and neck cancer samples, thus underlining the morphochemical conformity^6,37^. The following section will evaluate the influence of this pixelization effect on the optical resolution of the probe head.

### Investigations on the optical resolution of the fiber probe

**Figure 2:**
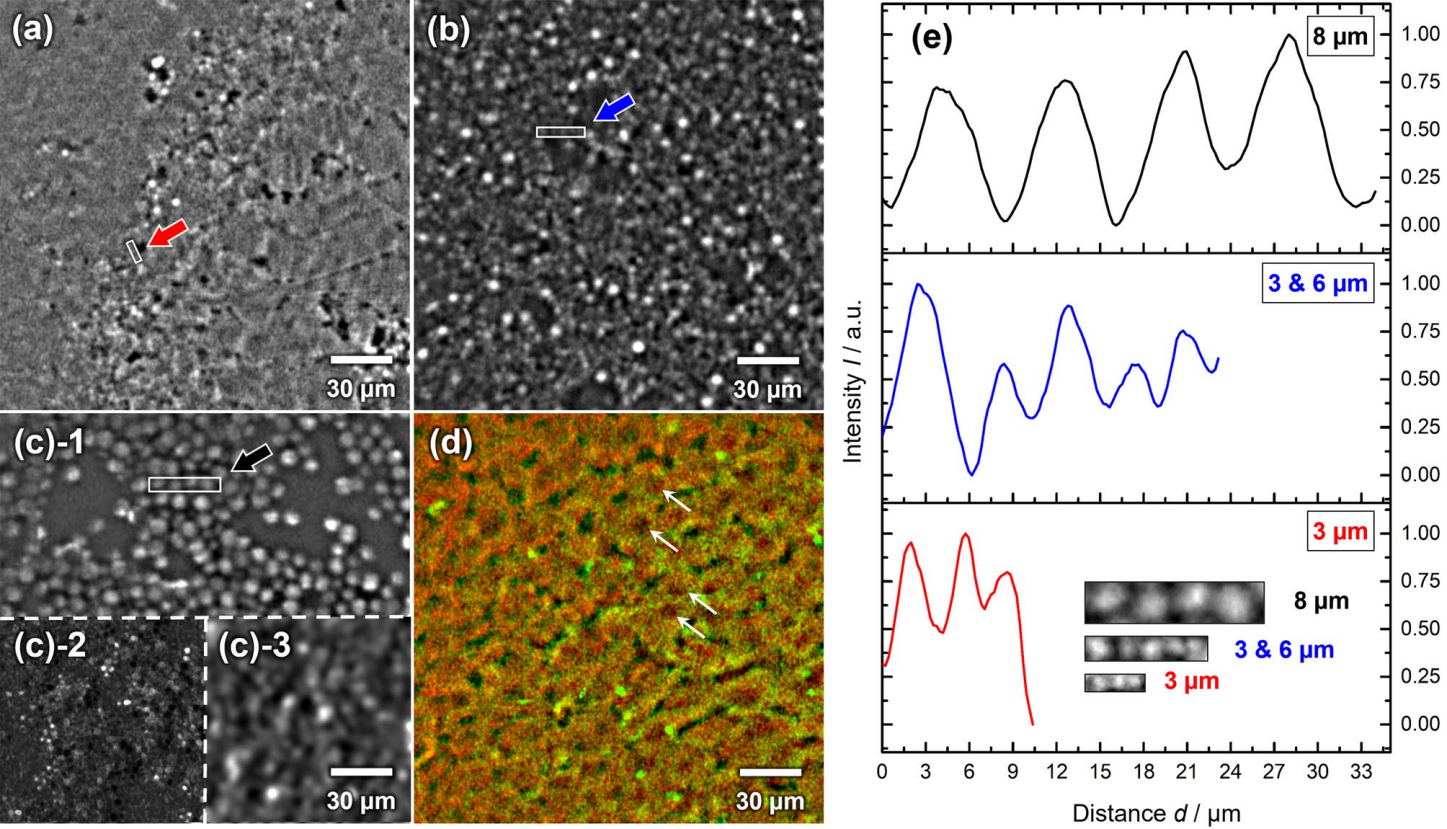
CARS images of (**a**) PS beads with a diameter of 3 µm, (**b**) Mixed PMMA and PS beads with diameters of 3 µm and 6 µm, respectively, (**c**) PS beads with a diameter of 8 µm. **(c)-1** superimposed image, **(c)-2** a single shot with normalization, **(c)-3** a single shot with normalization and an FFT bandpass filter. **(c)-1** to **(c)-3** are sections of the same field of view. (**d**) Mouse liver tissue cryo-section with a thickness of 20 µm (red: CARS, green: TPEF). (**e**) Normalized intensity profile of the indicated areas in (**a**–**c**) with averaging in the direction perpendicular to the rectangle’s long axes. Areas not covered by all 100 tiles in (**a**–**d**) were cropped. Final tile size: 210 × 210 µm^2^.

The comparison between the images recorded with the LSM and the fiber probe discussed in the previous section (see Fig. 1e vs. f) shows a limitation of the fiber probe, which is the pixelated image structure. In the following, we aim to evaluate the optical performance of the probe head concerning the influence of this pixelization on the spatial resolution within the focal plane. For this purpose, a polychromatic point spread function (PSF) was simulated regarding all optics incorporated within the probe head, excluding the imaging fiber. According to the simulation, an idealized point source at the object plane leads to an FWHM spot diameter of 1.6 µm in the image plane of the probe. To estimate the real focus size, the imaging fiber with its individual core diameters ranging from 2.2 to 3.7 µm must be considered. By convoluting the simulated PSF with the mean core diameter of 2.9 µm^38^, an actual focal spot size of 3.3 µm can be calculated. However, an according resolution can only be achieved by taking several images of the same sample with slightly shifted probe positions, i.e., less than the average core diameter between each image. To demonstrate the purely optical resolution limit of the fiber probe, CARS (2850 cm^−1^) images of clusters of polystyrene (PS) and polymethyl methacrylate (PMMA) beads with different diameters (3 µm, 6 µm, and 8 µm) and a liver tissue cryo-section (20 µm, mouse) were measured with a positional shift of 1 µm for 100 (10 by 10 tiles) images. The acquisition time for a single image was 8 s with a digital resolution of 1024 by 1024 pixels, a pixel dwell time of 2 µs, and four-times frame averaging. In Fig. 2, CARS images superimposed from 10 by 10 patterns of each sample show a dramatic improvement by reconstructing the space between the cores. It can be seen that 3 µm beads (Fig. 2a,b) are not well resolved with wavy patterns between neighboring objects in areas where they are densely packed while 8 µm beads (Fig. 2c) are readily resolved, as expected from the previous theoretical predictions. This becomes even more evident when looking at the intensity profiles (Fig. 2e) selected from the marked areas in Fig. 2a–c. Considering the inhomogeneous structure of the imaging due to different core sizes and spacing, densely packed beads, and non-resonant CARS background, it is challenging to give a specific value for the spatial resolution. However, Fig. 2e shows that the separation of two maxima is noticeably improving from 3 to 8 µm beads. Figure 2d demonstrates this approach for a histological sample, visualizing the liver’s unique sponge-like micro-architecture of alternating sinusoids (no/low contrast) and hepatocytes (high contrast). Densely sampling the plane by repetitive recording of spatially shifted images, in this case, brings the resolution to slightly subcellular levels. As a result, some nuclei become visible as dark round disks (white arrows, compare for example^39^, p. 232).

**Figure 3:**
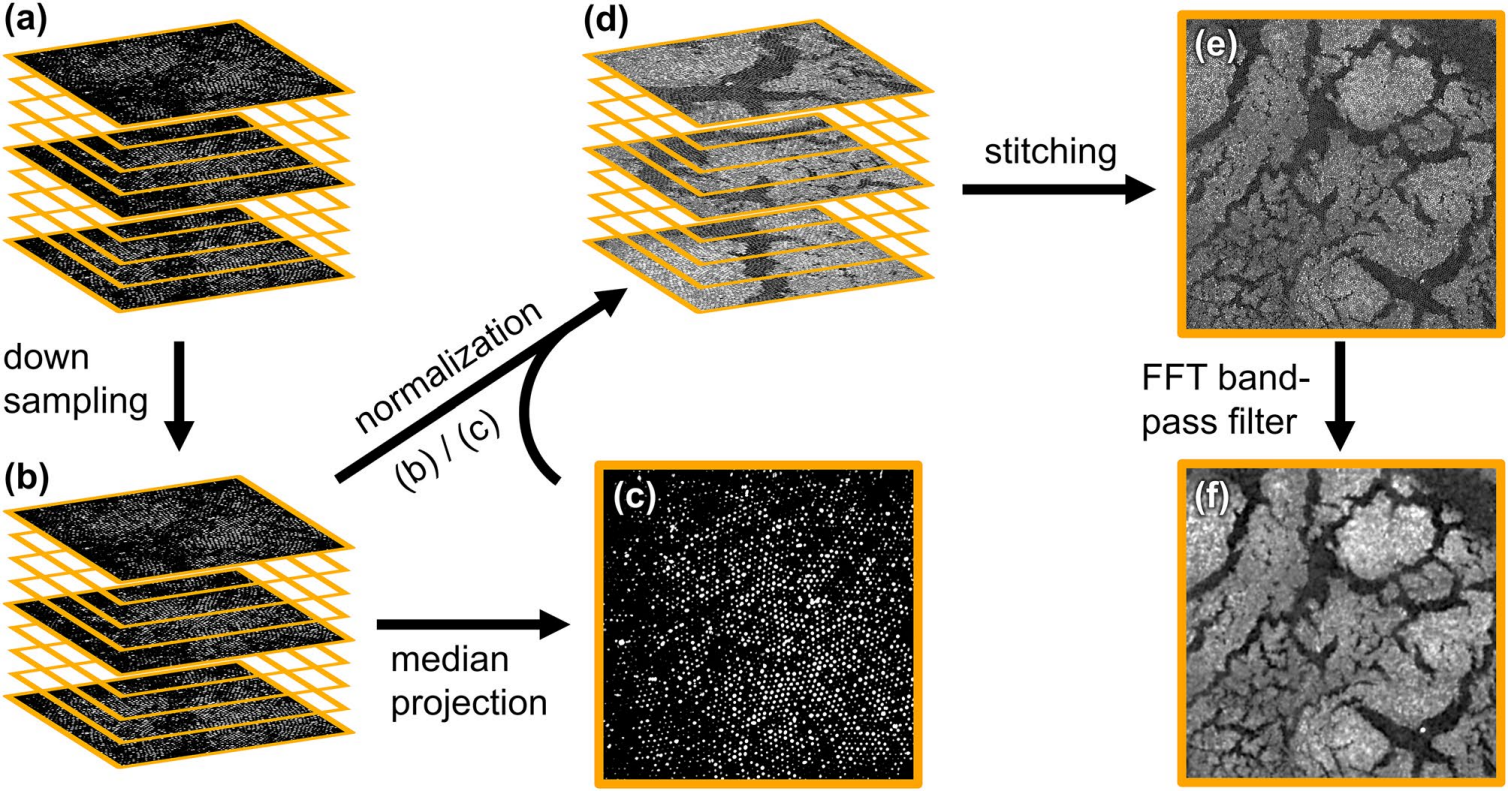
Image correction workflow. An area of the human head and neck tissue in Fig. 1 is used as an example. (**a**) Aligned images with corrected core positions throughout the stack. (**b**) Images are downscaled to a proper pixel scale to save computation time. (**c**) Median projection of the downscaled stack. (**d**) Stack divided by the median projection for normalization. (**e**) Stitched mosaic image using linear blending in overlapping areas. (**f**) Processed image after applying an FFT bandpass filter.

However, applying such a recording procedure would lead to a significant increase in the overall acquisition time not tolerable for in vivo measurements and, at the current stage, is, thus, mostly of theoretical interest. For individual images, the fiber structure and the spacing between the individual cores mostly determine the actual resolution of the fiber probe. With a mean core-to-core center distance of 4.6 µm and according to the sampling theorem by Nyquist and Shannon, the smallest periodical structure that the probe can resolve with a single shot has a pitch length of 2.3 * 4.6 ≈ 10.6 µm (ignoring the focal spot size). However, slight variance in core sizes, shapes, and distances (see also Fig. S2) would, locally, slightly change that number. Although this resolution may appear less competitive compared to the micron and sub-micron resolutions reported in other studies^18,35,40,41^ essential to note that this multicore fiber-based endoscope was conceptualized differently. Its primary objective is to facilitate nonlinear imaging at a tissue level without the need for electronic and moving parts in the endoscope. The estimated resolution of 10.6 µm is consistent with the experimental results, as can be seen by comparing a superimposed image (Fig. 2c-1) and a single image (Fig. 2c-2,c-3). It should be mentioned that it is likely close to optimal for the employed wavelengths as higher core densities lead to increased inter-core coupling. The FIGH-10-500N fiber used in this study strikes a balance between a relatively high core—and thus pixel—density, and keeping inter-core coupling to a minimum. In combination with a desirable degree of core structure non-uniformity, further minimizing the extent of inter-core coupling, it is particularly suited for our application^26,38^ and additionally helped by the fact that nonlinear modalities are generally less affected by weak inter-core coupling than linear ones. A detailed discussion is given in the SI, section S8. Since the presented probe design does not aim for examinations on a subcellular level but rather for distinguishing between different morpho-chemical structures on the tissue level, the resolution provided by the probe is sufficient for its purpose, as will be shown in the following sections. It should be mentioned, though, that, in a prospected hand-held operation mode and in combination with accurate positional tracking or real-time digital stitching, conditions leading to the above-described resolution enhancements may emerge quite naturally during (semi-)continuous scanning motions.

## Results and discussion

### Image processing

As mentioned above, the multi-core composition of the imaging fiber leads to a pixelated image structure (see Fig. 1d), which has two adverse effects: (1) the tissue morphochemistry is not well represented, making a qualitative judgment challenging; (2) the texture features used for image analysis approaches^6–8^ are strongly influenced by the pixelated structure making a quantitative analysis challenging. Therefore, post-processing steps are required to remove the core structure from the images and reconstruct the data. Ideally, this post-processing should allow for a correction of the transmission of individual fiber cores, ensuring comparability of different areas in one image as well as inter-probe comparability, and should calculationally not be too demanding to, perspectively, allow for online processing. There are three basic categories of established strategies for resolving the pixelation problem arising from imaging using coherent fiber bundles^42^: spatial averaging filters, spectral filtering, and interpolation methods. We found these established methods to yield unsatisfactory results and developed a custom methodology described below. A detailed qualitative comparison of the methods based on the work of Shinde and Matham^42^ is provided in the SI.

The reconstruction is achieved by the image correction workflow depicted in Fig. 3. As an example, 3 by 3 tiles from the upper right corner of the human head and neck tissue displayed in Fig. 1 are shown. In a first step, the original stack of images which has a shift of about one core-to-core center distance in 100 tiles due to varying laser coupling conditions undergoes a stack alignment procedure to ensure consistent fiber core positions in every image of the stack (Fig. 3a). After alignment, the stack is downscaled to reduce processing time in further calculations, preserving 2 * 2.3 pixels per core-to-core center distance (Fig. 3b). In a strictly periodic (orthogonal) structure, 2.3 pixels would be sufficient to sample the fiber core matrix according to the Nyquist criterion. The factor of 2 accounts for variations in the core diameters and imperfect core packing. Next, a median projection (Fig. 3c) of the downscaled stack is calculated to estimate the local efficiency of each fiber core. Every pixel value in every image of the downscaled stack is then divided by the corresponding pixel value of the median projection to normalize for the local changes in efficiency (Fig. 3d). This procedure takes the individual core transmittances into account without needing an external reference measurement. While this exact procedure requires raw data in the form of multiple images to be present, it should be noted that the median projection used for normalization does not necessarily have to stem from the same measurement, i.e., measurements of individual images are, of course, possible if an old median projection map is available. For practical applications, keeping a running median of the last 100 frames would be beneficial as this would lead to automatic calibration in the event of incoupling conditions slightly drifting over the course of the probe head’s lifetime.

The resulting images of the normalized stack are then stitched into one mosaic image using linear blending for overlapping areas (Fig. 3e). A magnification factor of 1.17 (sample site/incoupling site), probably due to imperfections in the alignment of optics in the redesigned probe head, was identified, and its influence on the tile overlap is taken into account during stitching. In the final step, an FFT bandpass filter is applied to eliminate any remaining core structure from the mosaic image (Fig. 3f). More detailed information on the image processing and motivations for the chosen parameters are provided in the SI.

Using appropriate parameters (e.g., overlap in tile stitching), the image reconstruction strategy shown in Fig. 3 was applied to all fiber probe images in this study, including a hollow organ tissue presented in the following section.

### Large surface imaging of bulky hollow organ tissue

The results presented in the preceding sections have shown that the fiber probe, in combination with image post-processing, yields multimodal images of thin tissue sections with a high quality representing the expected tissue morpho-chemistry. In the following, we aim to evaluate the capability of the fiber probe head for imaging large hollow organ areas, i.e., specimens of more than 10 mm^2^, as required during, e.g., endoscopy. For that purpose, a piece of small intestine tissue of a domestic pig (*Sus scrofa domestica*) and a native bulk human colon sample were used as test samples. The probe head was fixed by an appropriate mechanical assembly, while the sample could be moved in three axes relative to it by a motorized stage. With such a system, a reliable mosaic image acquisition can be achieved to visualize the morphochemistry of large surface areas of the tissue.

**Figure 4:**
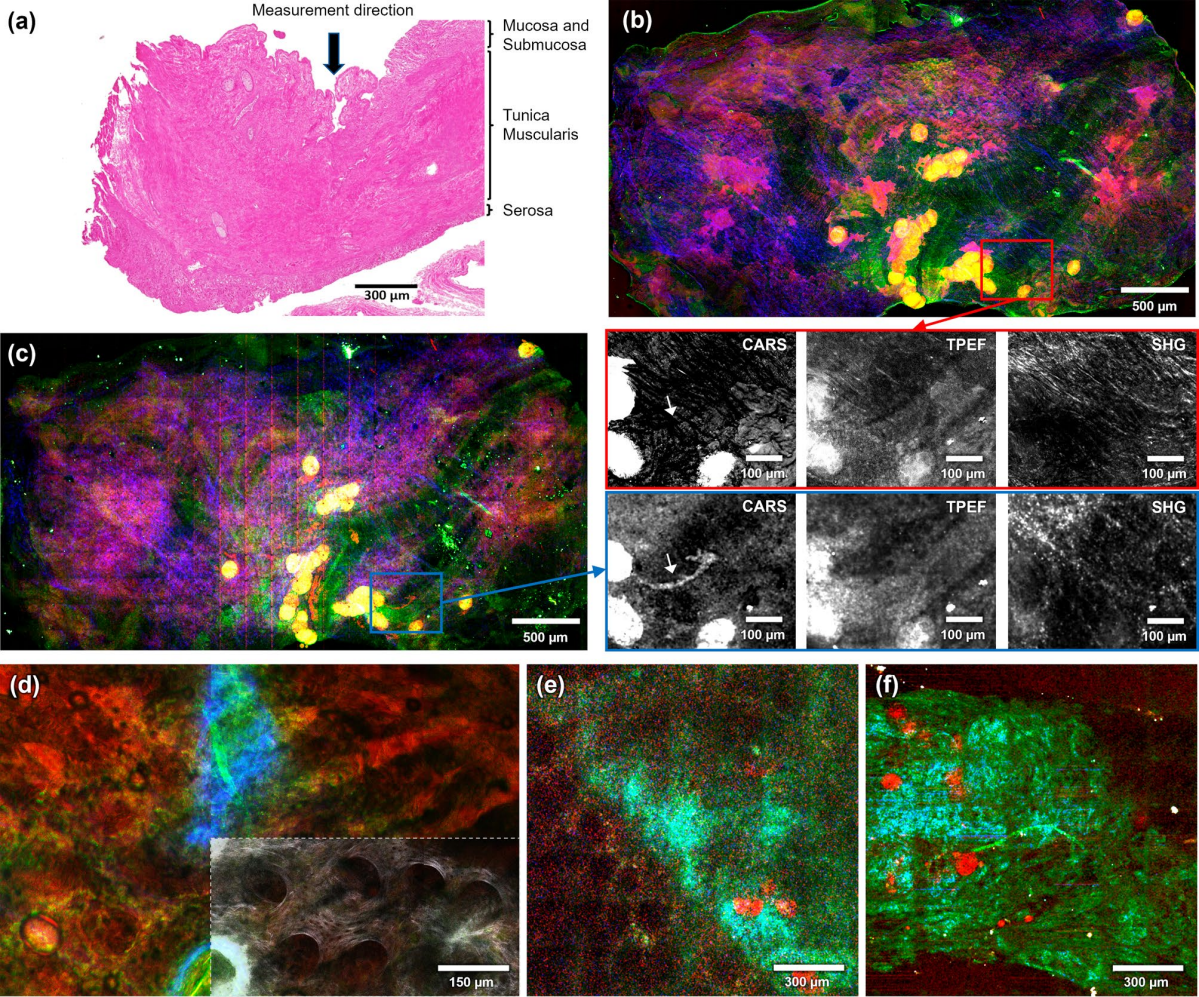
(**a**) H&E image of a pig small intestine section cut perpendicular to the intestine surface with histological-layer assignments. The arrow (black) indicates the measured layer (the rest of the mucosa and submucosa) for the LSM (**b**) and the probe recording (**c**). (**b**) LSM image of the inner surface of a pig intestine sample taken in the vicinity of (**a**). (**c**) Fiber probe image after image reconstruction. Tile size: 207 × 207 µm^2^. The marked areas, red in (**b**) and blue in (**c**), are enlarged in three separate channels for direct comparison of the same area. White arrows in CARS images of the enlarged views denote a dust-like object implying that the probe image was taken at a different focal plane, leading to a slightly different morphological contrast than the LSM image (**b**). (**d**) LSM image of inner surface of a bulk human colon. In the insert, a gray scale representation of the SHG channel with individually scaled brightness to account for the low intensities, compared to the blue regions in the main image, was overlayed (30% transparency) to highlight the crypt structures. (**e**) Fiber probe image of inner surface of a bulk human colon after image reconstruction. (**f**) Fiber probe image of inner colon surface with detached mucosa layer. Tile size in (**e**) and (**f**): 235 × 235 µm^2^. Color coding: red—CARS, blue—SHG, green—TPEF.

The inner part of the pig intestine, the mucosa layer, is routinely removed after slaughter as a preprocess of the following sausage production. The sample was put in phosphate-buffered saline to prevent it from drying out during large area scanning. The acquisition time for a single image in the pig intestine measurements was 32 s with a digital resolution of 2048 by 2048 pixels, a pixel dwell time of 2 µs, and 4 times averaging for both probe image and LSM image. Again, images were significantly oversampled, providing the ability to downsample the original data to emulate the effect of lower pixel resolutions. We found that reducing the pixel resolution to 256 by 256 pixels (corresponding to a frame acquisition time of ~ 0.5 s) does not lead to appreciable differences in the final result besides a slightly lower signal-to-noise ratio (Fig. S3). To cover a large area of the intestine tissue, 390 images (26 by 15 tiles, 190 µm lateral shift) with the probe and 77 images (11 by 7 tiles, 550 µm lateral shift) were recorded with the LSM and cropped to the sample area as shown in Fig. 4b,c. The bright vertical lines at the boundaries of the tiles in Fig. 4c are caused by an artifact due to a slight mismatch in the timing of the galvo mirror movement during scanning leading to a core position mismatch in those areas that negatively affects the normalization. For the bulk human colon measurements, the acquisition times for single images were 8 s and 16 s with pixel resolutions of 1024 by 1024 and 512 by 512, pixel dwell times of 2 µs and 4 µs, and 4 times averaging and 16 times averaging for Fig. 4e,f, respectively. The LSM image (Fig. 4d) was taken in a different LSM system (Zeiss LSM510, Ti:Sa laser (Coherent, USA) at 832 nm pumped by Neodymium-Vanadate CW laser, Optical Parametric Oscillator (APE, Germany) at 672.5 nm) with Zeiss Plan-Apochromat 20×/0.8 NA objective, a pixel resolution of 1024 by 1024, a pixel dwell time of 1.6 µs and 8 times averaging. Individual modalities were measured successively in epi-direction. The laser power at the sample was 40 mW for the pump beam (672.5 nm; TPEF, CARS) and 50/200 mW for the Stokes beam (832 nm; CARS/SHG, respectively). The LSM and probe images of bulk human colon were taken at different positions. The probe image in Fig. 4c shows high contributions of all three non-linear modalities. The morphochemistry displayed in these multimodal images was, again, histologically correlated with subsequent H&E staining. Figure 4a shows a cross-sectional H&E image of the pig intestine sample displayed in Fig. 4c, together with the main morphological features of an intestinal wall for structural conformation of the investigated sample. Typically, intestine samples have four distinct layers: mucosa, submucosa, tunica muscularis, and serosa. However, as mentioned above, most of the mucosa layer of the pig intestine was removed by the butcher. Therefore, in our sample, the first layer in the measurement direction was mainly comprised of submucosa and possibly remnants of the mucosa, as seen in the H&E image in Fig. 4a. The submucosa includes dense irregular layers of connective tissue with blood vessels, lymphatics, and nerves branching into the upper mucosa layer. A combination of strong signal coming from lipids (CARS in red) and collagen (SHG in blue) resulting in a violet color can be seen throughout the images in Fig. 4b,c. Intense, colocalizing CARS and TPEF signals (yellow) indicate distinct lipid accumulations that are well-visible in both images. Regions of strong TPEF signal (green) may originate from elastin in the submucosa and muscle layers below the submucosa. The overall resemblance of both images is excellent, indicating that, on the tissue level, equivalent information can be obtained.

The bulk human colon (Fig. 4d–f) still contains the mucosa layer. Accordingly, an LSM image (Fig. 4d) displays the typical crypt structures, as visible in the CARS (red) and TPEF (green) channels. As for the pig intestine sample, another feature apparent in the CARS channel is significant lipid accumulation. While similar accumulations are readily distinguishable in a corresponding fiber probe image (Fig. 4e, red spots), overall lower signal intensity and contrast in both CARS and TPEF makes the identification of crypts (bottom left) more difficult here. Cellular or subcellular features are not visible in either of the images. More broadly, the relative homogeneity of the mucosa at low resolutions in CARS/TPEF images appears to be a limiting factor. Structures in the submucosa and tunica muscularis that would provide strong SHG/TPEF are mostly out of focus in both, the LSM and fiber probe image. To demonstrate that this is an effect of the morphochemistry of the tissue rather than the probe itself, another fiber probe image was recorded in an area where the mucosa detached due to mechanical stress resulting from sample handling (Fig. 4f), showing the expected sharp features in the TPEF and SHG channel. Overall, and with the discussed limitations, Fig. 4 illustrates the capability of the presented multimodal CARS/SHG/TPEF probe to visualize the morphochemistry of hollow organs, which cannot be achieved by conventional microscopic techniques, e.g., white light endoscopy. The multimodal images show high morphologic conformity and image quality comparable in most regards to images obtained with a laser scanning microscope. No laser-induced photodamage was observed on the samples after imaging.

These results highlight the applicability of the presented probe design to medically relevant samples, specifically including bulk tissue. It is equally important, however, to highlight future steps still necessary towards an application-ready setup for clinical use case scenarios. These steps can be grouped into software- and hardware-related ones. An easy-to-clean surface at the distal fiber end is required for the hardware. As described above, for easier prototyping and more versatility in the testing phase, the current design does not include a fixed glass window and instead relies on a cover glass (170 µm) to be placed on the sample. As this limits the evaluation of a hand-held operating procedure and requires precise z-control by a mechanical fixture, the following design iteration will include a fixed window for operation under contact conditions. Also related to hand-held operation conditions is the currently achievable frame rate of about 2 fps (2 µs dwell time, 256 × 256 px; device limit: ~ 8 fps in unidirectional scanning mode) while retaining good image quality compared to Figs. 1, 4 (see also Fig. S3). Higher, i.e., true video, frame rates avoiding frame distortions during measurements under continuous probe movement or missing overlap between frames and resulting uncertainties in relative frame positions, are desirable and technically achievable by using a resonant scanner. In the current implementation, the scanning speed is still limited by the laser output power and detector sensitivity, both of which are addressable and unrelated to the probe design.

On the other hand, the need for multi-frame measurements could be partially eliminated by using a fiber that grants a larger field of view, such as the commercially available FIGH-100-1500N from Fujikura, which has a diameter of 1.5 mm and otherwise identical parameters as the fiber bundle used in this study. Currently, multi-frame stitching is carried out as a separate post-processing step and relies on the positional information provided by a microscope stage. Optical position sensors with the necessary precision are available. They could comparatively easily be integrated into the design, but software-based online stitching has also become feasible with modern hardware. Commercial solutions^43^ even account for motion and distortion artefacts, though not quite in an online manner. Such stitching requires true online image reconstruction which is our current focus regarding software-based improvements, as it is strictly necessary for the envisioned application scenario. These issues are non-fundamental and technical, resulting in a clear pathway for future developments towards a practical imaging device for real-time spectral histopathology that respects clinical requirements.

## Conclusion

This study demonstrated the potential of endoscopic multi-core non-linear imaging fiber probes for clinical applications. Multimodal CARS/SHG/TPEF images of bulk tissue samples taken with such a probe were shown to detail the morphochemistry of such samples at resolutions satisfying the requirements of the proposed application scenario while displaying excellent conformity with reference images taken on a conventional LSM. Furthermore, optimized design and better assembly quality compared to a previous implementation^32^ improved the optical performance, resulting in better illumination and increased signal collection efficiency. While we acknowledge that there will likely not be one single best endoscopic multimodal nonlinear imaging approach for all applications and, thus, technical development should be pushed forward for all implementations of the concept, our findings make multi-core fiber probes an appealing alternative for rigid endoscopes, scanning fiber endoscopes and miniaturized galvo scanning mirror-based or microelectromechanical systems for clinical applications. Specifically, multi-core fiber probes trade a simpler, more resilient, and potentially safer all-optical design without moving nor current carrying parts for a larger diameter, a lower laser power transmission and a loss in resolution as a consequence of their inherently pixelated images.

It was shown that the effects of this pixelization can be mitigated by appropriate image processing procedures. Furthermore, the processing procedure used in this study facilitates comparability of different areas in the same image and between images taken with different probes which will be relevant for future work, especially in the context of a clinical application. Finally, the next technical steps for future developments towards practical imaging devices for a number of fields, including dermatology, gastroenterology, neuro, or head and neck surgery, are discussed. In combination with suitable image analysis algorithms, the discussed fiber probe design can establish a fast, convenient, and label-free spectral histopathology approach without the need to remove tissue, avoiding potential risks and inconveniences inherent to classical histopathological and, thus, biopsy-based approaches.

### Biological samples

The native bulk human colon sample and the human head and neck cancer sample were received after surgery from the University Hospital Jena. The subjects gave informed consent and the study was approved by the ethical committees at the University Hospital Jena. All methods were carried out in accordance with the relevant guidelines and regulations as per the Declaration of Helsinki. The pig (*Sus scrofa domestica*) intestine sample was commercially purchased from a local butcher store (Fleischerei Hönnger, Jena).

### Supporting information

Digital supporting information is available online and includes a detailed description of all data processing steps for image reconstruction, including a respective flowchart, a summary of all relevant reconstruction parameters, a detailed description of the reconstruction algorithm, an overview of the process for core-to-core distance determination, an image quality comparison after reconstruction for different original digital resolutions, details on the signal-to-noise approximation, a discussion of different depixelation approaches, and a discussion of design considerations regarding core-to-core cross-talk and resolution. In addition, the image reconstruction algorithm itself is available as a separate file.

### Supplementary Information

Supplementary Information 1.

Supplementary Information 2.

## Data Availability

Original data are available from the corresponding author upon reasonable request.
