# Supplementary material for: Feasibility studies of multimodal nonlinear endoscopy using multicore fiber bundles for remote scanning from tissue sections to bulk organs
Source: Sci Rep. 2023 Aug 23;13:13779. doi: 10.1038/s41598-023-40944-6 (PMC10447453; doi:10.1038/s41598-023-40944-6)
Supplement: Supplementary file 1 — Supplementary Information 1. [file 41598_2023_40944_MOESM1_ESM.docx]

/*

In the following the exact processing procedure and the meaning of all user adjustable parameters will be explained. For better clarity the images and stacks of images occurring in the various processing steps will be numbered. The macro was written for FIJI 1.52p.

Data formats and input data structure:

The macro expects the input data to be stored as individual images, organized in a separate folder for each channel. The user is prompted to input the parent directory of those channel folders. The macro will try to deduce the correct order of images from their names using the Array.sort() function. Data formats supported by default are ‘.tif’, ‘.Tif’, ‘.TIF’, ‘.tiff’, ‘.Tiff’, ‘.TIFF’, and ‘.lsm’ but any format supported by FIJI may be added if needed.

User adjustable parameters:

Upon executing the macro the user will be presented with a window requesting relevant parameters. These parameters are

‘core center distance / px’,

‘core center distance / µm’,

‘sampling factor’,

‘FFT-factor’,

‘n of tiles in X’,

‘n of tiles in Y’,

‘tile overlap in %’,

'mapping factor probe side / LSM side',

‘stitching type’,

‘stitching direction’,

‘tile blending’,

‘output bit depth’.

The effect these parameters have will be discussed below.

Reading the input data:

The individual channels will be processed sequentially, saving on memory. First the macro will read all files from the first folder (corresponding to the first channel) inside the specified directory. Any subfolders inside this channel folder will be ignored. The files will be sorted alphabetically and organized in a stack (1).

Data processing:

Before any processing is done, the original stack is converted into a 32 bit stack (2) as to retain maximum numerical precision throughout the processing. Assuming some oversampling in the raw data, in a first processing step the images are downsampled speeding up all further processing steps. The scaling factor (xnew=xold*scaling factor, ynew=yold*scaling factor) is calculated according to ‘scaling factor’=2.3/‘core center distance / px’*‘sampling factor’ giving the user the freedom to choose the amount of oversampling by varying the ‘sampling factor’. This results in a downsampled stack (3). Choosing a ‘sampling factor’ of 1 will result in the average core-to-core distance being represented by 2.3 pixels, according to the Nyquist criterion. We recommend a ‘sampling factor’ of 2 for smoother looking images which in our case also addresses the fact that there is a distribution of nearest core-to-core distances rather than one single value. The downsampling is done using bilinear interpolation (slightly reducing time requirements as compared to bicubic interpolation and avoiding halo effects due to overshooting).

From the downsampled stack (3) a median projection (4) is calculated and stack (3) is then divided by that median projection, resulting in a normalized stack (5). The median projection, as compared to an average, has the advantage that mostly black background areas have a smaller influence. The median projection is assumed to reflect the core specific characteristics like transmission characteristics in a way that is to some degree independent of the specific sample. For this approach to work best of course a sufficiently large stack of raw images is required in which more than half of the measured area is occupied by signal producing sample. While this approach is obviously not perfect it doesn’t require difficult to obtain external reference data like actual transmission profiles. The macro doesn’t provide any option to use an external reference for the normalization step but such an option can easily be included if needed.

In the next step the stack is transformed into a mosaic (6) using the built-in plugin ‘Grid/Collection stitching’ and the initially specified parameters ‘tile overlap in %’, ‘stitching type’, ‘stitching direction’, ‘tile blending’. The ‘Grid/Collection stitching’ plugin requires every tile to be stored in a separate file in one common folder. Because of that the individual slices of stack (4) are saved to the tmp folder and deleted again after the stitching. Several tile blending methods are available but usually ‘linear blending’ yields the best results. N.B.: The 'mapping factor' is not used to correct the 'tile overlap' and its influence on the 'tile overlap' has to be taken into account manually. The 'mapping factor' is only used to calculate the correct scale for the final image.

The mosaic (6) is then converted into an image of the specified ‘output bit depth’ (7). For most purposes the original bit depth option is sufficient.

In the final processing step an FFT filter is applied rejecting features of a size less than 2.3 px*’sampling factor’*’FFT-factor’ (autoscale and saturate options in ImageJ enabled). This blends the individual cores and the cladding, avoiding the impression of an unrealistically high resolution. Setting the FFT-factor to 0 results in a non-filtered output image showing the original core structure. An FFT-factor of 1 is usually the best choice.

After all channels have been processed to mosaics, they are joined into one stack (9). If more than one channel was processed but less than four, they are color coded and transformed into a composite image according to rg (for two channels) or rgb (for three channels), otherwise all channels will be left grey. The coloring of channels is handled slightly differently in a specific case: if all channel names contain either the string "CARS" or "TPEF" or "SHG" (not case sensitive) and none of those strings appear twice, a specific color scheme is applied (CARS: red, TPEF: green, SHG: blue), according to a group internal convention.

Output:

The result stack (9) will be saved under the name ‘Composite.tif’ in the channels parent folder along with a .txt file containing all parameters specified by the user as well as the scaling factor. The individual slices of stack (9) will be saved as grey scale images in an output folder ‘out’ in the respective channel folders under the name ‘Stitched_image.tif’.

This macro was written by Marko Rodewald, Friedrich Schiller University Jena, 2020

*/

//############################################################################################

// Requesting parameters and the processing folder, writing parameters to file

//--------------------------------------------------------------------------------------------

parameters=newArray("","","","","","","","");

Dialog.create("Parameters");

Dialog.addNumber("core center distance", 38.3547104, 8, 10, "px");

Dialog.addNumber("core center distance", 4.62751682, 8, 10, "µm");

Dialog.addNumber("sampling factor", 2);

Dialog.addNumber("FFT-factor", 1);

Dialog.addNumber("n of tiles in X", 12);

Dialog.addNumber("n of tiles in Y", 12);

Dialog.addNumber("tile overlap in % (two-sided)", 8, 8, 10, "");

Dialog.addNumber("mapping factor probe side / LSM side", 1.168410373, 8, 10, "");

Dialog.addChoice("stitching type", newArray("Grid: row-by-row", "Grid: column-by-column", "Grid: snake by rows", "Grid: snake by columns"), "Grid: row-by-row");

Dialog.addChoice("stitching direction", newArray("Right & Down", "Left & Down", "Right & Up", "Left & Up"), "Right & Up");

Dialog.addChoice("tile blending", newArray("Linear Blending", "Average", "Median", "Max. Intensity", "Min. Intensity", "Intensity of random input tile"),"Linear Blending");

Dialog.addChoice("output bit depth", newArray("8-bit", "16-bit", "32-bit", "original"), "original");

Dialog.show();

for (i = 0; i<parameters.length; i++) {

parameters[i] = Dialog.getNumber();

}

core_center_distance_px=parameters[0];

core_center_distance_mum=parameters[1];

sampling_factor=parameters[2];

scaling_factor=2.3/core_center_distance_px*sampling_factor;

f_factor=parameters[3];

n_tilesX=parameters[4];

n_tilesY=parameters[5];

tile_overlap=parameters[6];

mapping_factor=parameters[7];

stitching_type=Dialog.getChoice();

stitching_direction=Dialog.getChoice();

tile_blending=Dialog.getChoice();

output_bit_depth=Dialog.getChoice();

parent_dir = getDirectory("Choose a Directory ");

files=getFileList(parent_dir)

channels=newArray();

var mosaic_image_size_x=0;

file=File.open(parent_dir+"\\parameters.txt");

print(file, "core to core distance / px: "+parameters[0]);

print(file, "core to core distance / µm: "+parameters[1]);

print(file, "sampling factor: "+parameters[2]);

print(file, "scaling factor: "+scaling_factor);

print(file, "FFT-factor: "+parameters[3]);

print(file, "number of tiles in x: "+parameters[4]);

print(file, "number of tiles in y: "+parameters[5]);

print(file, "tile overlap in %: "+parameters[6]);

print(file, "mapping factor probe side / LSM side: "+parameters[7]);

print(file, "stitching type: "+stitching_type);

print(file, "stitching direction: "+stitching_direction);

print(file, "tile blending: "+tile_blending);

print(file, "output bit depth: "+output_bit_depth);

File.close(file);

//############################################################################################

print(" ");

print("#############################");

print("Starting image reconstruction");

print("#############################");

//############################################################################################

// Finding subfolders marking different channels, calling the reconstruction function for

// each channel

//--------------------------------------------------------------------------------------------

for (i = 0; i < files.length; i++) {

if (endsWith(files[i],"/")) {

channels=Array.concat(channels, files[i]);

}

}

print("Processing the following folders:");

for (i = 0; i < channels.length; i++) {

print(channels[i]);

}

for (i = 0; i < channels.length; i++) {

reconstruction(channels[i]);

}

//############################################################################################

//############################################################################################

// Specifying the reconstruction function

//--------------------------------------------------------------------------------------------

function reconstruction(channel){;

setBatchMode(true);

run("Misc...", "divide=0 run");

print("#############################");

print("Processing folder "+channel);

dir=parent_dir+channel; //specifying the respective directory for each channel

list = getFileList(dir); //listing all files in respective channel folder

list = Array.sort(list); //sorting the file list alphabetically so that no very specific file naming format is neccessary

a=0;//initiate image counter a

for (i = 0; i < list.length; i++) {

if (endsWith(list[i], "/")==1 && endsWith(list[i], "out/")==0){//ignoring subfolders (all ending with "/")

print("Ignoring "+list[i]);

}

else {

path = dir + list[i]; //creating a path variable to adress each file individually

if (endsWith(path, ".lsm") || endsWith(path, ".Tif") || endsWith(path, ".TIF") || endsWith(path, ".TIFF") || endsWith(path, ".Tiff") || endsWith(path, ".tiff") || endsWith(path, ".tif")) {//checks for file endings to select only images in the .lsm or .tiff format. More file endings can easily be added here. The checking is case sensitive (unfortunately).

open(dir + list[i]); //opens each image in the channel folder

rename(channel+a); //renames each image to give it the name of the channel + consecutive numbers

//print(channel+a);

if (a!=0) {

run("Concatenate...", "open image1="+channel+" image2="+channel+a); //adds every newly opened image to a stack of all images opened before

}

rename(channel); //renames the updated stack back to "channel" in order for the concatenate function to operate properly

a++; //increases the image counter

}

}

}

//########################################################################################

// Checking if the specified number of tiles matches the number of slices in the stack

// returns an error and exits the macro if the numbers don't match

//----------------------------------------------------------------------------------------

rename("Stack_original");

if (nSlices!=n_tilesX*n_tilesY) {

exit("Number of files ("+nSlices+") in "+dir+" does not match tile specifications ("+n_tilesX+"*"+n_tilesY+")");

}

// determining the input bit depth for output option ("original bit depth")

if (output_bit_depth=="original") {

output_bit_depth=toString(bitDepth())+"-bit";

}

//########################################################################################

//########################################################################################

// Calculate final mosaic size in µm - necessary at this stage to avoid numerical artifacts

// due to scaling

//----------------------------------------------------------------------------------------

tile_size_x=getWidth()/core_center_distance_px*core_center_distance_mum/mapping_factor;

mosaic_image_size_x=tile_size_x*n_tilesX-(n_tilesX-1)*tile_overlap/100*tile_size_x;

//########################################################################################

//########################################################################################

// Any preprocessing steps applied to the whole stack may be inserted here

//----------------------------------------------------------------------------------------

run("32-bit"); //improves numerical precision

//run("Median...", "radius=3 stack");

//########################################################################################

//########################################################################################

// Downscaling of oversampled images with

// scaling_factor=2.3/core_center_distance_px*sampling_factor

// the 2.3 in the above expression is the nyquist criterium

// the downscaling makes most common preprocessing steps like Gaussian blurring, filtering

// etc. unnecessary

//----------------------------------------------------------------------------------------

run("Scale...", "x="+scaling_factor+" y="+scaling_factor+" z=1.0 interpolation=Bilinear average process create");

rename("Stack_scaled");

selectWindow("Stack_original");

run("Close");

//########################################################################################

//########################################################################################

// Making a median projection of the scaled stack for normalization purposes

//----------------------------------------------------------------------------------------

selectWindow("Stack_scaled");

run("Z Project...", "projection=Median");

rename("Median_projection");

//########################################################################################

//########################################################################################

// Divide each image in the scaled stack by the median projection. To avoid numerical

// inaccuracies, 32 bits are used for the output of this operation

//----------------------------------------------------------------------------------------

imageCalculator("Divide create 32-bit stack", "Stack_scaled","Median_projection");

selectWindow("Result of Stack_scaled");

rename("Stack_scaled_normalized");

//########################################################################################

//########################################################################################

// Tidying up a bit by closing unneccessary images

//----------------------------------------------------------------------------------------

selectWindow("Stack_scaled");

run("Close");

selectWindow("Median_projection");

run("Close");

//########################################################################################

//########################################################################################

// Create a temporary directory, save the normalized slices to it individually

//----------------------------------------------------------------------------------------

selectWindow("Stack_scaled_normalized");

//-------------------------------------------------------

// additional operations to be done to the normalized stack before stitching may be specified here

//-------------------------------------------------------

tmp=getDirectory("temp")+"temp_tiles_"+getTime()+"\\"; // creates a unique path to avoid any possible confusion if several instances of imagej are run at the same time

File.makeDirectory(tmp);// creates a temporary directory to which all individual images of the stack are stored. This step is only neccessary becaus the stitching procedure requires a folder with all images to be stitched in it. The folder will be deleted later on. Also, physically storing the images makes it possible to close the stack image before stitching to free some RAM.

run("Image Sequence... ", "format=TIFF digits=3 save="+tmp+"Stack_scaled_normalized000.tif");

run("Close");// closes the normalized stack

//########################################################################################

//########################################################################################

// The actual stitching is performed

//----------------------------------------------------------------------------------------

run("Grid/Collection stitching", "type=["+stitching_type+"] order=["+stitching_direction+"] grid_size_x="+n_tilesX+" grid_size_y="+n_tilesY+" tile_overlap="+tile_overlap+" first_file_index_i=0 directory="+tmp+" file_names=Stack_scaled_normalized{iii}.tif output_textfile_name=TileConfiguration.txt fusion_method=["+tile_blending+"] regression_threshold=0.30 max/avg_displacement_threshold=2.50 absolute_displacement_threshold=3.50 compute_overlapcompute_overlapsubpixel_accuracy display_fusion computation_parameters=[Save computation time (but use more RAM)] image_output=[Fuse and display]");

//run("Select Bounding Box (guess background color)");

//run("Crop");

rename("Stitched_image");

//########################################################################################

//########################################################################################

// Deleting all temporarily created images from the temporary folder and the folder itself

//----------------------------------------------------------------------------------------

temp_list = getFileList(tmp);

for (i=0; i<temp_list.length; i++) {

ok = File.delete(tmp+temp_list[i]);

}

ok = File.delete(tmp);

//########################################################################################

//########################################################################################

//Adjusts the output bit-depth to the desired value. Afterwards a fft bandpass filter is

//applied. If these operations are performed in the opposite order, the autoscale and

//saturate options of the fft filter don't work properly leading to poor usage of the

//target number space.

//----------------------------------------------------------------------------------------

selectWindow("Stitched_image");

//run("Enhance Contrast...", "saturated=0.1 normalize"); // only interesting if dealing with 32-bit images

run(output_bit_depth);

largest_dimension=maxOf(getWidth(), getHeight());

run("Bandpass Filter...", "filter_large="+largest_dimension+" filter_small="+2.3*sampling_factor*f_factor+" suppress=None tolerance=5 autoscale saturate");

//########################################################################################

//########################################################################################

//Creates an output folder in the channels directory and saves the stitched image to it.

//----------------------------------------------------------------------------------------

File.makeDirectory(dir+"out\\");

saveAs("tif", dir+"out\\Stitched_image.tif");

run("Close");

//########################################################################################

}

//############################################################################################

setBatchMode(false);

print("...");

//############################################################################################

//Opens the stitched images of every channel and renames them to the respective channel folder

//name.

//--------------------------------------------------------------------------------------------

for (i = 0; i < channels.length; i++) {

open(parent_dir+channels[i]+"out/Stitched_image.tif");

rename(substring(channels[i], 0, lengthOf(channels[i])-1)); //-1 omitts the final "/"

run("Set Scale...", "distance="+getWidth()+" known="+mosaic_image_size_x+" pixel=1 unit=µm");

}

//############################################################################################

//############################################################################################

//Count how many channels contain either "CARS" or "TPEF" or "SHG" as a string.

//--------------------------------------------------------------------------------------------

b=0;

for (i = 0; i < channels.length; i++) {

if (matches(channels[i], "(?i).*CARS.*") || matches(channels[i], "(?i).*TPEF.*") || matches(channels[i], "(?i).*SHG.*")) {

b=b+1;

}

}

//############################################################################################

//############################################################################################

//Check if "CARS", "TPEF", and "SHG" each appear only once

//--------------------------------------------------------------------------------------------

nCARS=0//number of channels containing "CARS" as a string

nTPEF=0//number of channels containing "TPEF" as a string

nSHG=0//number of channels containing "SHG" as a string

for (i = 0; i < channels.length; i++) {

if (matches(channels[i], "(?i).*CARS.*")){

nCARS=nCARS+1;

}

if (matches(channels[i], "(?i).*TPEF.*")){

nTPEF=nTPEF+1;

}

if (matches(channels[i], "(?i).*SHG.*")){

nSHG=nSHG+1;

}

}

//############################################################################################

//############################################################################################

//If all channels contain either "CARS" or "TPEF" or "SHG" and none of those strings appear

//twice, a specific color scheme is applied (CARS-red, TPEF-green, SHG-blue)

//--------------------------------------------------------------------------------------------

if (b==channels.length && nCARS<=1 && nTPEF<=1 && nSHG<=1 && channels.length>1) {

run("Images to Stack", "name=Stack title=[] use");

run("Make Composite", "display=Composite");

for (i = 1; i < channels.length+1; i++) {

setSlice(i);

if (matches(getInfo("slice.label"), "(?i).*CARS.*")) {

run("Red");

}

if (matches(getInfo("slice.label"), "(?i).*TPEF.*")) {

run("Green");

}

if (matches(getInfo("slice.label"), "(?i).*SHG.*")) {

run("Blue");

}

}

saveAs("tif", parent_dir+"Composite.tif");

}

else {

if (channels.length>1) {

run("Images to Stack", "name=Stack title=[] use");

if (channels.length<4) {

run("Make Composite", "display=Composite");

saveAs("tif", parent_dir+"Composite.tif");

}

}

}

//############################################################################################

print("done");

exit();
