# Supplementary material for: Feasibility studies of multimodal nonlinear endoscopy using multicore fiber bundles for remote scanning from tissue sections to bulk organs
Source: Sci Rep. 2023 Aug 23;13:13779. doi: 10.1038/s41598-023-40944-6 (PMC10447453; doi:10.1038/s41598-023-40944-6)
Supplement: Supplementary file 2 — Supplementary Information 2. [file 41598_2023_40944_MOESM2_ESM.docx]

Supplementary Information (SI)

Feasibility Studies of Multimodal Nonlinear Endoscopy Using Multicore Fiber Bundles for Remote Scanning from Tissue Sections to Bulk Organs

Hyeonsoo Bae,^a,b,c^ Marko Rodewald,^a,b^ Tobias Meyer-Zedler,^a,b^ Thomas W. Bocklitz,^b^ Gregor Matz,^d^ Bernhard Messerschmidt,^d^ Adrian T. Press,^c,e,f^ Michael Bauer,^c,e^ Orlando Guntinas-Lichius,^g^ Andreas Stallmach,^h^ Michael Schmitt,^b^ and Juergen Popp*^,a,b^

^a^Leibniz Institute of Photonic Technology (Leibniz IPHT), Member of Leibniz Health Technologies, Member of the Leibniz Centre for Photonics in Infection Research (LPI), PO Box 100239, Jena, Germany, 07702

^b^Friedrich-Schiller University Jena, Institute of Physical Chemistry and Abbe Center of Photonics, Helmholtzweg 4, Jena, Germany, 07743

^c^Center for Sepsis Control & Care (CSCC), Jena University Hospital, Erlanger Allee 101, Jena, Germany, 07747

^d^GRINTECH GmbH, Schillerstraße 1, Jena, Germany, 07745

^e^Jena University Hospital, Department of Anesthesiology and Intensive Care Medicine, Am Klinikum 1, Jena, Germany, 07747

^f^Friedrich-Schiller University Jena, Medical Faculty, Kastanienstr. 1, 07747 Jena

^g^Jena University Hospital, Department of Otorhinolaryngology, Am Klinikum 1, Jena, Germany, 07747

^h^Jena University Hospital, Department of Internal Medicine IV, Am Klinikum 1, Jena, Germany, 07747

**1. Data Processing: General Overview**

The data processing can be divided into three separate steps: a) determining the average core-to-core center distance and the core-to-core center distance distribution which are important for choosing reasonable sampling parameters, b) stack alignment of the raw data and c) the image reconstruction. The following scheme gives an overview of all relevant steps.


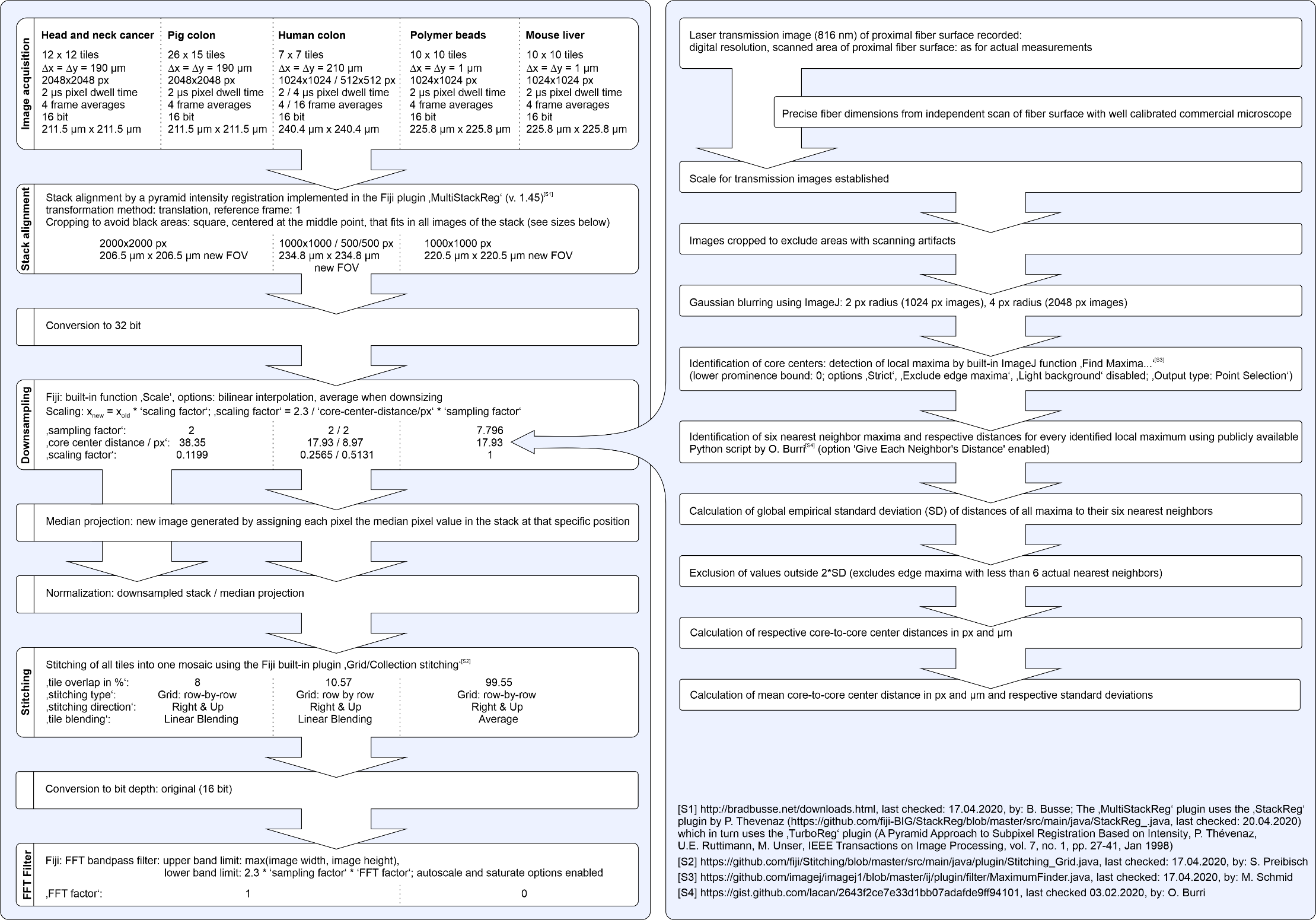


**Figure S1.** Overview of all relevant data processing steps.

**2. Determining the Core-to-Core Center Distance**

Determining the average core-to-core center distance and the core-to-core center distance distribution is important for choosing reasonable sampling parameters. To do so, a laser transmission image (816 nm) was recorded for every set of measurement parameters (digital resolution, scanned area of the proximal fiber surface) used in the actual measurements. As opposed to using an image recorded in one of the three non-linear modalities, CARS, SHG, and TPEF, this approach has the benefit that it is independent of any specific sample and the amount of signal the sample gives at the position of any given fiber core. Transmission measurements had to be performed only once unless the above-mentioned measurement parameters were changed. Slightly different alignment requirements between measurement days made it necessary to record two such images for two sets of measurement parameters, having 2048×2048 px (pig colon, head and neck cancer measurements) and 1024×1024 px (polymer bead measurements) respectively.

Knowing the precise fiber dimensions from an independent scan of the fiber surface with a well-calibrated commercial microscope, a precise scale could be applied to those transmission images. The images were cropped to exclude any areas with scanning artifacts and slightly smoothed by Gaussian blurring using ImageJ (v. 1.52p) and a radius of 4 px (2048×2048 px image) and 2 px (1024×1024 px image) respectively. The core centers represent local maxima and were detected using the built-in ImageJ function ‘Find Maxima…’ using a lower prominence bound of 0 with the options ‚Strict‘, ‚Exclude edge maxima‘, ‚Light background‘ disabled [https://github.com/imagej/imagej1/blob/master/ij/plugin/filter/MaximumFinder.java by M. Schmid, last checked: 17.04.2020]. In the next step, the six nearest neighbors of every core and the respective distances in pixels (with subpixel accuracy) and micrometers of unique pairs were determined using a publicly available python script for the 2D-K-Nearest-Neighbors algorithm by O. Burri with the option ‘Give Each Neighbor’s Distance’ enabled [https://gist.github.com/lacan/2643f2ce7e33d1bb07adafde9ff94101, last checked 03.02.2020].

Naively using the resulting values would, however, overestimate the average core-to-core distance *d*, because cores close to the edge or to a blind core will have one or several closest neighbors within the group of 6 nearest neighbors that is not representative of the otherwise quite dense packing. Therefore, the empirical standard deviation of all values was calculated and all values outside twice this standard deviation around the estimated mean were excluded from the data set. An adjusted mean core-to-core center distance *d*_adj_ and an adjusted standard deviation s_adj_ were calculated from the trimmed data set. The resulting standard errors of *d*_avg,adj_ were 0.002% (2048×2048 px image) and 0.004% (1024×1024 px image). The fact that the adjusted estimated standard deviations are quite large (about 4% of the average core-to-core distance) has implications on the resolution which becomes a function of x and y. The actual values for *d*_avg,adj_ used in the image reconstruction algorithm were specified with higher numerical precision than the ones specified in figure S1 to avoid rounding errors adding up over the many thousand pixels in large tiled images.

| 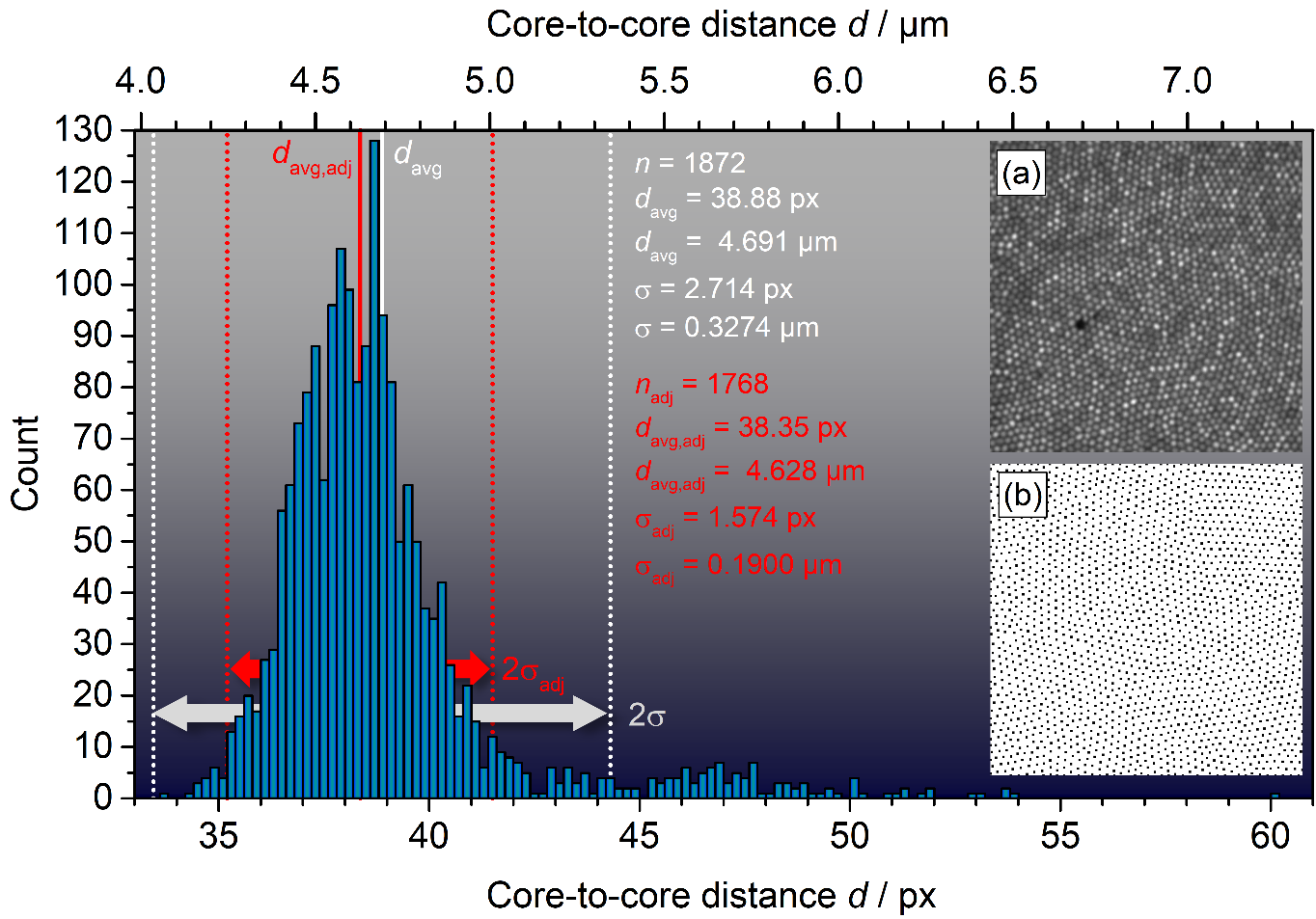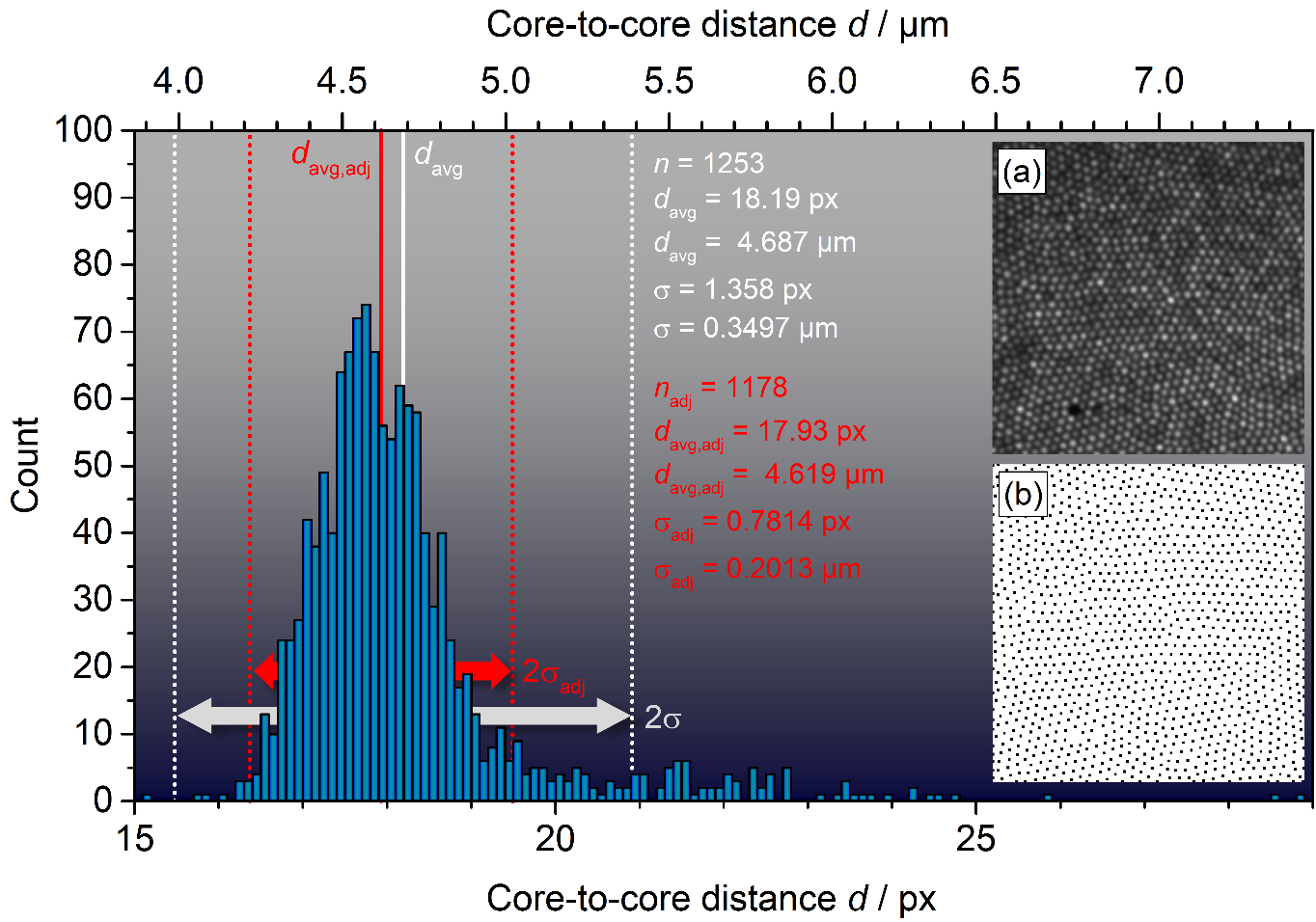 |
| --- |

**Figure S2.** Core-to-core center distance distributions between all unique pairs of cores for which the partnering cores are within the group of their respective 6 nearest neighbors. Top: for the pig colon and head and neck cancer tissue measurements (bin size: 0.2 px); bottom: for the polymer bead measurements (bin size: 0.1 px). Inserts (a) show the analyzed part of the fiber surface, inserts (b) the identified local maxima represented as black squares and corresponding to core center positions.

**3. Image Reconstruction**

The image reconstruction is done using an ImageJ (FIJI) macro (also available in the supporting information). In this section, the exact processing procedure and the meaning of all user-adjustable parameters will be explained in detail. For better clarity, the images and stacks of images occurring in the various processing steps will be numbered. The macro was written for FIJI 1.52p.

**Data formats and input data structure:**

The macro expects the input data to be stored as individual images, organized in separate folders for each channel. The user is prompted to input the parent directory of those channel folders. The macro will try to deduce the correct order of images from their names using the *Array.sort()* function. Data formats supported by default are ‘.tif’, ‘.Tif’, ‘.TIF’, ‘.tiff’, ‘.Tiff’, ‘.TIFF’, and ‘.lsm’ but any format supported by FIJI may be added if needed.

**User-adjustable parameters:**

Upon executing the macro the user will be presented with a window requesting relevant parameters. These parameters are ‘*core center distance / px’, ‘core center distance / µm’, ‘sampling factor’, ‘FFT-factor’, ‘n of tiles in X’, ‘n of tiles in Y’, ‘tile overlap in %’, ’mapping factor probe side/LSM side’, ‘stitching type*’, ‘*stitching direction*’, ‘*tile blending*’, ‘*output bit depth*’. The effect these parameters have will be discussed along the way.

**Reading the input data:**

The individual channels will be processed sequentially, saving on memory. First, the macro will read all files from the first folder (corresponding to the first channel) inside the specified directory. Any subfolders inside this channel folder will be ignored. The files will be sorted alphabetically and organized in a stack (1).

**Data processing:**

Before any processing is done, the original stack is converted into a 32-bit stack (2) as to retain maximum numerical precision throughout the processing. The data sets presented in this paper were recorded with quite severe oversampling providing maximum flexibility regarding any data processing. Therefore, in the first processing step, the images are downsampled speeding up all further processing steps. The scaling factor (x_new_=x_old_*scaling factor, y_new_=y_old_*scaling factor) is calculated according to ‘scaling factor’=2.3/‘*core center distance / px*’*‘*sampling factor*’ giving the user the freedom to choose the amount of oversampling by varying the ‘*sampling factor*’. This results in a downsampled stack (3). Choosing a ‘*sampling factor*’ of 1 will result in the average core-to-core distance being represented by 2.3 pixels, according to the Nyquist criterion. We recommend a ‘*sampling factor*’ of 2 for smoother-looking images which in our case also addresses the fact that there is a distribution of nearest core-to-core distances rather than one specific value. The downsampling is done using bilinear interpolation (slightly reducing time requirements as compared to bicubic interpolation and avoiding halo effects due to overshooting).

From the downsampled stack (3) a median projection (4) is calculated to estimate the local efficiency and stack (3) is then divided by that median projection, resulting in a normalized stack (5). The basic assumption is that each core sees a similar brightness distribution of the multiple sample images in a stack. The median projection, as compared to an average, has the advantage that mostly black background areas have a smaller influence. The median projection is assumed to reflect the core-specific characteristics like transmission efficiency in a way that is to some degree independent of the specific sample. For this approach to work best, of course a sufficiently large stack of raw images is required in which more than half of the measured area is occupied by signal-producing sample. While this approach is obviously not perfect it doesn’t require difficult-to-obtain external reference data like actual transmission profiles. The macro doesn’t provide any option to use an external reference for the normalization step, but such an option can easily be included if needed.

In the next step, the stack is transformed into a mosaic (6) using the built-in plugin *‘Grid/Collection stitching’* [https://github.com/fiji/Stitching/blob/master/src/main/java/plugin/Stitching_Grid.java by S. Preibisch, last checked: 17.04.2020] and the initially specified parameters ‘*tile overlap in %*’, ‘*stitching type*’, ‘*stitching direction*’, ‘*tile blending*’. The *‘Grid/Collection stitching’* plugin requires every tile to be stored in a separate file in one folder. Because of that the individual slices of stack (5) are saved to the tmp folder and deleted again after the stitching. For the colon and the head and neck cancer data sets ‘linear blending’ was chosen as the tile blending method (polymer beads and mouse liver tissue: ‘Average’). N.B.: The *'mapping factor'* is not used to correct the *'tile overlap'* and its influence on the *'tile overlap'* has to be taken into account manually (more convenient for our specific workflow). The *'mapping factor'* is only used to calculate the correct scale for the final image.

The mosaic images (6) bit depth is then changed according to the users ‘output bit depth’ selection (7). For all data sets presented in this paper the original bit depth of 16 bit was chosen.

In the final processing step an FFT filter is applied rejecting features of a size less than 2.3 px*’sampling factor’*’FFT-factor’ (autoscale and saturate options in ImageJ enabled). This amalgamates the individual cores and the cladding, avoiding the impression of an unrealistically high resolution. Setting the FFT-factor to 0 results in a non-filtered output image showing the original core structure. For the colon and the head and neck cancer data an FFT-factor of 1 was chosen (polymer beads, mouse liver tissue: 0). The resulting image (8) is stored in an output folder ‘out’ inside the respective channel folder.

After all channels have been processed into mosaics, they are joined into one hyperspectral stack (9). If more than one channel was processed but less than four, they are color-coded and transformed into a composite image according to rg (for two channels) or rgb (for three channels), otherwise, all channels will be left grey. The coloring of channels is handled slightly differently in a specific case: if all channel names contain either the string "CARS" or "TPEF" or "SHG" (not case sensitive) and none of those strings appear twice, a specific color scheme is applied (CARS: red, TPEF: green, SHG: blue), according to a group-internal convention.

**Output:**

The resulting stack (9) will be saved under the name ‘Composite.tif’ in the channels parent folder along with a .txt file containing all parameters specified by the user as well as the scaling factor. The individual slices of stack (9) will be saved as greyscale images in an output folder ‘out’ in the respective channel folders under the name ‘Stitched_image.tif’.

**4. Processing Parameters**

**5. Resolution Comparison**


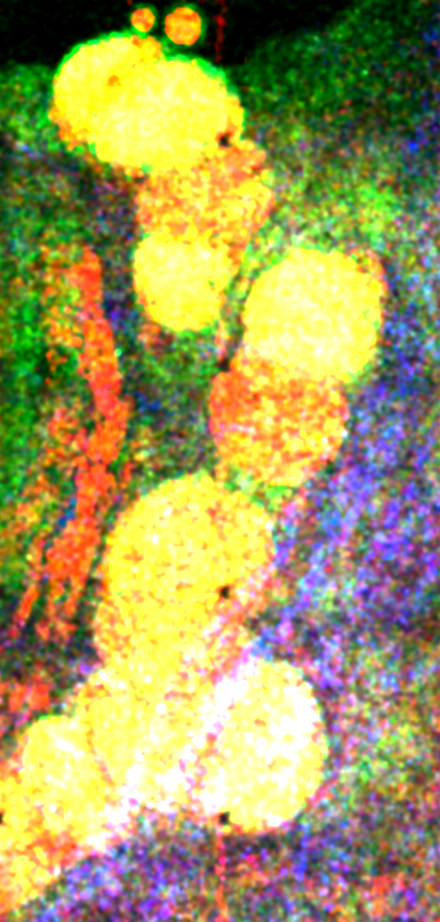

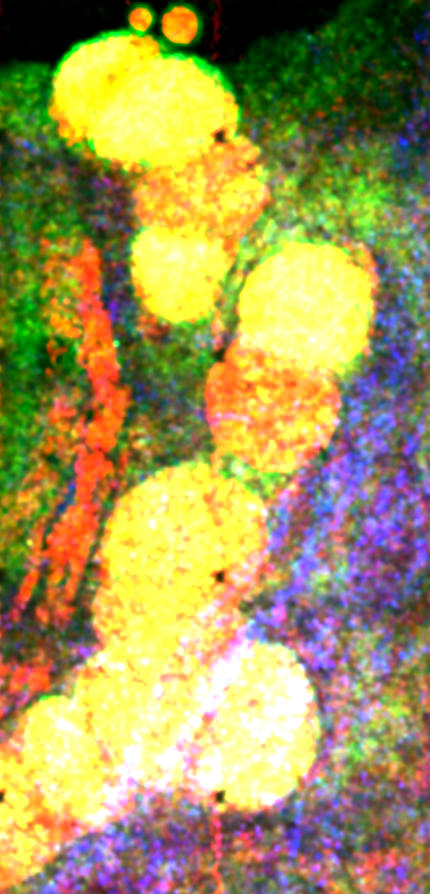

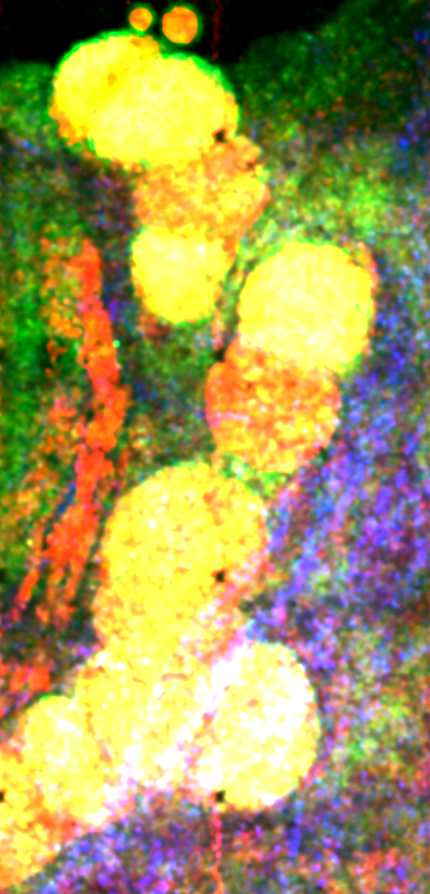

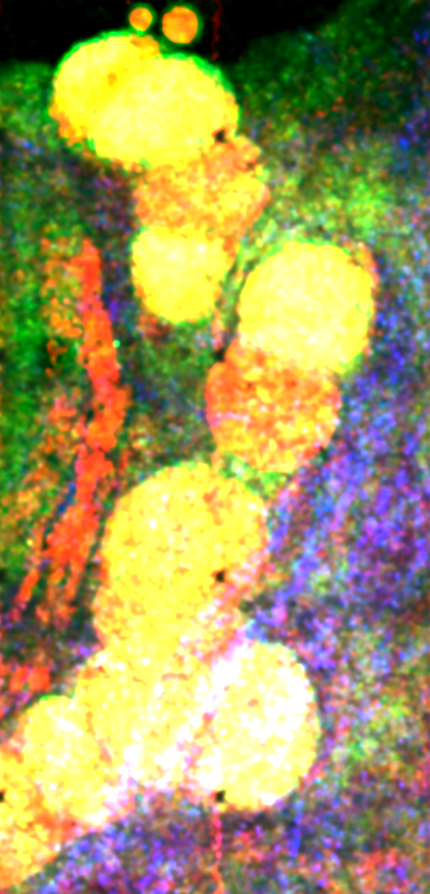


**Figure S3.** Quality comparison between images of different pixel resolution after reconstruction. Digital tile resolutions from left to right: 256x256 px, 512x512 px, 1024x1024 px, 2048x2048 px. Smaller images are subsampled from the largest-resolution image stack (successive removal of every other pixel; no binning/averaging applied) and, thus, show very slightly offset views. Displayed is an enlarged view of a region at the bottom-center of Fig 4(c). Observable quality differences are minimal and only manifest in slightly worse signal-to-noise ratios in images with lower pixel resolution.

**6. Signal-to-noise-ratio approximation for Fig. 1(g)**

The signal-to-noise ratio is very dependent on the specific sample under investigation and the amount of signal it generates, as well as on the respective core. It thus becomes a strongly spatially and sample-dependent quantity and cannot be considered a general instrument parameter as each core would have to be considered a separate “instrument”. However, to provide an example and a rough approximation: If the noise is estimated as the standard deviation in the cladding region of an unprocessed image (which assumes that the noise is independent of the signal intensity), the peak signal-to-noise ratio (pSNR) can be expressed as the (bit depth (16 bit) minus the mean noise level) divided by the noise SD, as detector gains were typically adjusted to give a few pixels per image with overflow. If this is done for the central tile depicted in Fig. 1(j) (4 frame averages), the pSNR(4) becomes ~103 for CARS, ~22 for TPEF, and ~23 for SHG. Under the assumption that this value increases with Sqrt(n), pSNR(1) values of 52, 11, and 11 for CARS, TPEF, and SHG, respectively can be estimated. Note that the pSNR for CARS includes the non-resonant background.

**7. Comparison of depixelation strategies**

There are three basic categories of strategies for resolving the pixelation problem arising from imaging using coherent fiber bundles (i.e. removing the ‘comb structure’) [S5]: spatial averaging filters, spectral filtering, and interpolation methods. Spatial averaging using uniform (circular/quadratic) kernels or non-uniform Gaussian averaging filters are well known for leading to blurry images with reduced contrast as they conceptually represent low-pass filters (i.e. they lose relevant higher-frequency information) [S5]. Spectral filtering using hand-crafted masks applied to the 2D Fourier transform of the image to eliminate characteristic patterns in the frequency domain image originating from the semi-regular core-grid pattern, represents an improvement over simple spatial averaging but is not free of problems either. As opposed to spatial averaging, this method allows to be somewhat more selective with regards to the frequencies to be filtered out. However, both, spatial averaging filters and spectral filtering, have to use rather generous parameters/masks (meaning strong smoothing) to sufficiently hide the cladding, as well as the non-uniform brightness distribution within images of individual cores. The need for carefully handcrafted masks for spectral filtering is another problem of that approach and even if great care is taken, undesirable artefacts, e.g. ringing, typically remain. Deviations from a perfectly regular comb pattern exacerbate this problem. After extensive testing, we found both approaches to yield unsatisfactory results. It is noteworthy that neither of those two methods intrinsically takes specific fiber characteristics like differing core transmittivities into account (though this could, in principle, be achieved in a similar way to the method described in the manuscript by considering multiple frames).

For the above-described reasons, we therefore - internally - used an interpolation method before the development of the method described in the manuscript. Interpolation methods aim to identify the individual core positions, extract the central intensities, and interpolate values between the sparse sampling points using a suitable interpolation function (like Delaunay triangulation or natural neighbor interpolation). They rely on a good reference image for the identification of core positions which, as a positive side effect, also enables intensity calibration. A conceptually different approach to identifying individual core positions in densely sampled images is to only address previously identified individual cores in the first place (so-called ‘fiber-core-targeted scanning’), skipping the outer core regions as well as the cladding, as demonstrated by Li et al. [S6]. Some of the practical limitations of interpolation-based approaches are well summarized by Shinde and Matham [S5]:

‘Localizing the center pixels for fiberlet, finding natural neighbours for every pixel and calculat-
ing natural neighbour coordinates is time consuming. Ideally it needs to be done only once, but in practice there are changes in position of proximal end of fiber bundle relative to camera; stressing the need for recalibration. In practice calibration needs to be done after every few frames resulting in delayed signal display, it hamper frame rate of the flexible endoscopic system.’

Again, a suitable reference image could be obtained in a similar way to the method described in the manuscript by considering multiple frames, however, the processing time demands are potentially problematic for our future goal of online processing. Additionally, we observed undesirable Voronoi-type structures from the interpolation in the resulting images which were visually disturbing and could not easily be dealt with. Regarding any type of subsequent automated image analysis, these would be similarly problematic as the original comb patterns.

For those reasons, we opted to use the method described in the manuscript which consists of a two-step procedure: 1) normalization (takes different core transmittivities into account, eliminates non-uniform intensity distribution within the cores, is agnostic towards irregularities in the comb pattern); 2) modest (and fine-tunable) Fourier filtering. The latter does not have to deal with the whole comb pattern (as opposed to the above-discussed Fourier-based method) but mostly with the remaining cladding lines which are much thinner than any resolvable sample fine structure. This leads to sample detail being visually better preserved. Additionally, depending on chosen filtering strength, it slightly blends the (after normalization) mostly intensity-uniform core patches.

**8. Design considerations regarding core-to-core cross-talk and resolution**

Crosstalk between cores is an important aspect of multi-core imaging fibers to consider, especially regarding the achievable resolution. The imaging fiber used in our fiber probe (FIGH-10-500N) was carefully selected, taking its crosstalk characteristics into consideration. However, it should be noted that crosstalk is much less problematic for nonlinear imaging modalities than for linear ones because of the higher-order dependence of the signal from the incident beam intensity. As an example, one might consider TPEF or SHG: taking into account only the intensity and assuming everything else equal, even if the 6 surrounding cores of any given core were to acquire 5% each of the total intensity coupled into the central core, the resulting signal would still be heavily dominated by that generated from light transmitted by the central core (98:3 –> 0.7²:(6*(0.05)²)). Two relevant features to consider when it comes to core-to-core coupling are core density and illumination wavelength. However, it is known that nonuniformity in the structures of coherent fiber bundles suppresses the efficiency of crosstalk to neighboring cores and, therefore, plays an important role as well. These effects were investigated in detail by two studies, 1) "Numerical analysis of light propagation in image fibers or coherent fiber bundles [S7]" and 2) "Experimental and theoretical analysis of core-to-core coupling on fiber bundle imaging [S8]".

Both studies compared the imaging fibers FIGH-10-500N (10000 cores, 600 µm fiber diameter, 4.5 µm core spacing, 2.9 µm core diameter, 10-15% core size variation) and FIGH-10-350S (10000 cores, 450 µm fiber diameter, 3.2 µm core spacing, 2 µm core diameter, 7-10% core size variation) with identical specifications except for core-to-core distance and core diameter.

The two imaging fibers, and cross-coupling more generally, were characterized and quantified in great detail in the above-mentioned papers but to summarize: longer wavelengths and larger core densities lead to more cross-coupling, while increased core shape and size variation greatly reduces the amount of cross-coupling, meaning that a balance has to be struck for optimal resolution. In our case, the wavelengths were fixed (816 nm and 1064 nm), leaving the other factors open as design decisions. Transmission images of a United States Air Force (USAF) target taken by scanning a CW laser over the input face of the imaging fiber bundle lead the authors of 2) to the following conclusion:

‘Because fiber bundle 350S has a smaller core diameter and spacing than 500N, fiber bundle 350S was expected to have a better resolution than fiber 500N. However, our results in Fig. 5 show that fiber 500N can resolve the air force target better than 350S, particularly at the longer illumination wavelengths (978 nm). Our results show that the imaging performance of a fiber bundle is determined not only by the core density, but also by the core-to-core coupling strength. The denser the fiber bundle is, the higher the number of potentially resolvable pixels; on the other hand, high core density can result in strong inter-core coupling, significantly reducing the “effective pixel numbers”.’

Considering this context, we carefully selected the imaging fiber (FIGH-10-500N) with a moderate variation of core size and spacing to achieve a desirable amount of nonuniformity of core structures, while still providing an appropriate core density, leading to an overall close to optimal resolution within the wavelength constraints.

**9. References**

[S1] http://bradbusse.net/downloads.html, last checked: 17.04.2020, by: B. Busse; The ‚MultiStackReg‘ plugin uses the ‚StackReg‘ plugin by P. Thevenaz (https://github.com/fiji-BIG/StackReg/blob/master/src/main/java/StackReg_.java, last checked: 20.04.2020) which in turn uses the ‚TurboReg‘ plugin (A Pyramid Approach to Subpixel Registration Based on Intensity P. Thévenaz, U.E. Ruttimann, M. Unser IEEE Transactions on Image Processing, vol. 7, no. 1, pp. 27-41, Jan 1998).

[S2] https://github.com/fiji/Stitching/blob/master/src/main/java/plugin/Stitching_Grid.java, last checked: 17.04.2020, by: S. Preibisch

[S3] https://github.com/imagej/imagej1/blob/master/ij/plugin/filter/MaximumFinder.java, last checked: 17.04.2020, by: M. Schmid

[S4] https://gist.github.com/lacan/2643f2ce7e33d1bb07adafde9ff94101, last checked 03.02.2020, by: O. Burri

[S5] Shinde, Anant; Matham, Murukeshan Vadakke (2014): Pixelate Removal in an Image Fiber Probe Endoscope Incorporating Comb Structure Removal Methods. In: J Med Imaging Hlth Inform 4 (2), S. 203–211. DOI: 10.1166/jmihi.2014.1255.

[S6] Li, Qian; Rohringer, Wolfgang; Preißer, Stefan; Erkkilä, Mikael T.; Haindl, Richard; Sattmann, Harald et al. (2021): Depixelation of coherent fiber bundle imaging by fiber-core-targeted scanning. In Appl. Opt. 60 (26), pp. 7955–7962. DOI: 10.1364/AO.430537.

[S7] Reichenbach, Kristen Lantz; Xu, Chris (2007): Numerical analysis of light propagation in image fibers or coherent fiber bundles. In Opt. Express 15 (5), pp. 2151–2165. DOI: 10.1364/OE.15.002151.

[S8] Chen, Xianpei; Reichenbach, Kristen Lantz; Xu, Chris (2008): Experimental and theoretical analysis of core-to-core coupling on fiber bundle imaging. In Opt. Express 16 (26), pp. 21598–21607. DOI: 10.1364/OE.16.021598.
